# The age of adult pilocytic astrocytoma cells

**DOI:** 10.1038/s41388-021-01738-0

**Published:** 2021-03-17

**Authors:** Natalia Voronina, Christian Aichmüller, Thorsten Kolb, Andrey Korshunov, Marina Ryzhova, Jill Barnholtz-Sloan, Gino Cioffi, Martin Sill, Andreas von Deimling, Stefan M. Pfister, Jan Gronych, David T. W. Jones, Jonas Frisén, Marc Zapatka, Aurélie Ernst

**Affiliations:** 1 Group Genome Instability in Tumors, DKFZ, Heidelberg, Germany; 2 Division of Molecular Genetics, DKFZ, DKFZ-Heidelberg Center for Personalized Oncology (HIPO) and German Cancer Consortium (DKTK), Heidelberg, Germany; 3 Department of Neuropathology, Heidelberg University Hospital, and CCU Neuropathology, DKFZ, Heidelberg, Germany; 4 NN Burdenko Neurosurgical Institute, Moscow, Russia; 5 Department of Population and Quantitative Health Sciences, Case Western Reserve University School of Medicine, Cleveland, OH USA; 6 Central Brain Tumor Registry of the United States, Hinsdale, IL USA; 7 Hopp Children’s Cancer Center Heidelberg (KiTZ); Division of Pediatric Neurooncology, DKFZ; German Cancer Consortium (DKTK), DKFZ; Department of Pediatric Oncology, Hematology & Immunology, Heidelberg University Hospital, Heidelberg, Germany; 8 Hopp Children’s Cancer Center Heidelberg (KiTZ), Division of Pediatric Neurooncology, DKFZ; German Cancer Consortium (DKTK), DKFZ, Heidelberg, Germany; 9 Hopp Children’s Cancer Center Heidelberg (KiTZ), Pediatric Glioma Research Group, Heidelberg, Germany; 10 Department of Cell and Molecular Biology, Karolinska Institute, Stockholm, Sweden; 11 Present Address: Roche Diagnostics Deutschland GmbH, Mannheim, Germany

**Keywords:** CNS cancer, Cancer genetics

## Abstract

Adult pilocytic astrocytomas (PAs) have been regarded as indistinguishable from pediatric PAs in terms of genome-wide expression and methylation patterns. It has been unclear whether adult PAs arise early in life and remain asymptomatic until adulthood, or whether they develop during adulthood. We sought to determine the age and origin of adult human PAs using two types of “marks” in the genomic DNA. First, we analyzed the DNA methylation patterns of adult and pediatric PAs to distinguish between PAs of different anatomic locations (*n* = 257 PA and control brain tissues). Second, we measured the concentration of nuclear bomb test-derived ^14^C in genomic DNA (*n* = 14 cases), which indicates the time point of the formation of human cell populations. Our data suggest that adult and pediatric PAs developing in the infratentorial brain are closely related and potentially develop from precursor cells early in life, whereas supratentorial PAs might show age and location-specific differences.

## Introduction

Pilocytic astrocytomas (PAs) are the most frequent brain tumor in children. In adults, however, PAs occur much less commonly than other types of primary brain tumors, such as glioblastomas or meningiomas. Although there is some evidence of differential localization and of a distinct spectrum of MAPK alterations in adult PAs [1, 2], there is currently no clear consensus regarding differences in other large-scale molecular patterns by age. It is currently unknown whether adult PAs arise early in life but do not lead to symptoms and thus detection before adulthood, or whether they develop during adult life. In the latter case, adult PAs could originate either from adult precursor cells or from late hits in precursor cells similar to the cells of origin of pediatric PAs. If adult PAs arise from early precursor cells, as their pediatric counterparts, it will be critical to understand why they are detected so late. If adult PAs develop during adulthood, it will be important to identify differences in the molecular features and the biological behavior of these tumors that distinguish them from pediatric PAs. Therefore, we sought to determine the age and origin of adult PAs. We used two types of “marks” in the tumor cell genomic DNA to address this question.

First, DNA methylation patterns of tumor cells typically reflect the cell of origin of a tumor [3–5]. Besides somatically acquired alterations in the DNA methylome, brain tumor epigenomes maintain DNA methylation signatures of the cells giving rise to these tumors and can be used to accurately classify central nervous system tumors [6]. Analyses of methylation patterns in PAs previously showed that the most significant differences between tumors are linked to the brain regions where these tumors develop [7, 8]. Age-related differences in PA methylomes have been described to a very limited degree. Individual analyses for each brain region have not been performed, to identify potential age-related differences that would otherwise not be visible due to the tight association between tumor location and methylation patterns. In children, methylation profiles of PAs have been linked to tumor behavior. DNA methylation profiles specific to PA location and predictive of clinical behavior were reported recently [9]. However, this study did not include adult PA patients and was limited to discrimination between infants and non-infants. In a cohort of 20 pediatric PAs, distinct methylation profiles characterizing PAs with onset before or after 3 years of age were identified [10]. This finding, together with the lack of studies focusing on adult patients, highlights the importance of identifying further potential age-related differences in PAs.

In addition to distinguishing between PAs of different origins based on DNA methylation patterns, it is possible to assess the age of tumor cells. Measuring the concentration of nuclear bomb test-derived ^14^C in genomic DNA indicates the time point of the generation of human cell populations [11]. Retrospective birth-dating of cells has been applied to numerous cell types in humans, such as neurons [12, 13], oligodendrocytes [14], cardiomyocytes [15] and adipocytes [16]. This approach provides unique opportunities to address the question of cell turnover and determine the age of human cells. The rationale behind the method is that when a cell goes through mitosis, it integrates ^14^C in the synthesized genomic DNA. The concentration of integrated ^14^C corresponds to that in the atmosphere, creating a date mark in the DNA. The accuracy of individual datings is approximately ±2 years, with variations depending on the date of the sample collection and the date of birth of the individual [11]. Using retrospective birth-dating, the growth dynamics of benign meningiomas were described, showing that these tumors are resected on average two decades after their time of origin [17].

Making use of these two “marks” in nucleic acids, ^14^C in genomic DNA and DNA methylation patterns, we sought to determine the age and origin of adult PA cells.

## Results

**Fig. 1:**
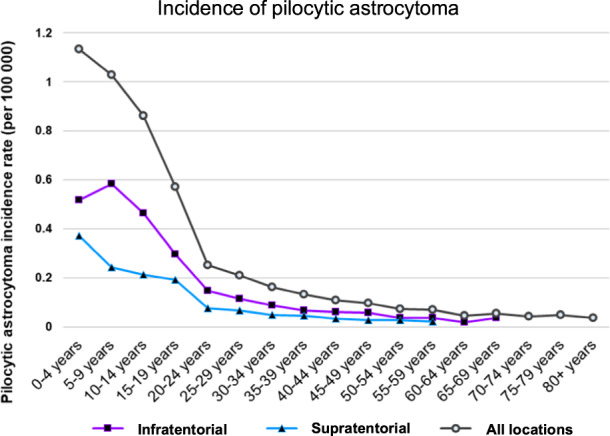
Age-adjusted incidence rates of pilocytic astrocytomas (age 0–69 years, NPCR and SEER data 2012–2016). The incidence is shown separately for pilocytic astrocytomas located in the supratentorial (blue) or infratentorial (purple) brain regions. The gray line indicates the total incidence for all pilocytic astrocytomas, including those for which no information on the location was available. Rates are per 100,000 and age-adjusted to the 2000 US Std Population (age groups according to Census P25-1130) standard, unless the number of reported cases is less than 16 (e.g., after 59 years for supratentorial cases and after 69 years for infratentorial cases).

To get insights into the timing of PA development, we first analyzed PA incidence rates across age groups. Certain brain tumors, such as Sonic Hedgehog medulloblastomas, are known to occur in a bimodal age distribution, making up the majority of infant and adult medulloblastomas, but only a minor fraction of childhood tumors [18]. In the case of Sonic Hedgehog medulloblastomas, this bimodal age distribution suggests the existence of heterogeneity between pediatric and adult tumors [18]. For PAs, however, there is only one early age peak of incidence that shows a sharp decline down to the age of 20 years (Fig. 1), hinting towards less heterogeneity between adult and pediatric PAs, as compared with other tumors that have two distinct incidence peaks. Importantly, the decrease in incidence rates with increasing age holds true for both infratentorial and supratentorial PAs (Fig. 1). It will be important to understand when adult PAs develop and from which cells they originate.

DNA methylation analyses can be used to identify molecular tumor subtypes and get insights into the tumor cell of origin [6]. Previous studies analyzing methylation patterns in PAs showed that the most significant differences between tumors are linked with the brain regions where these tumors develop [7, 8]. To identify potential age-related differences that would otherwise not be detectable due to the tight association between tumor location and methylation patterns, we analyzed separately infratentorial and supratentorial PAs. In total, the cohort comprised 121 supratentorial and 136 infratentorial tissue samples (adult, adolescent and pediatric PAs as well as control brain tissues for each brain region, see Supplementary Table 1) analyzed on methylation arrays.

**Fig. 2:**
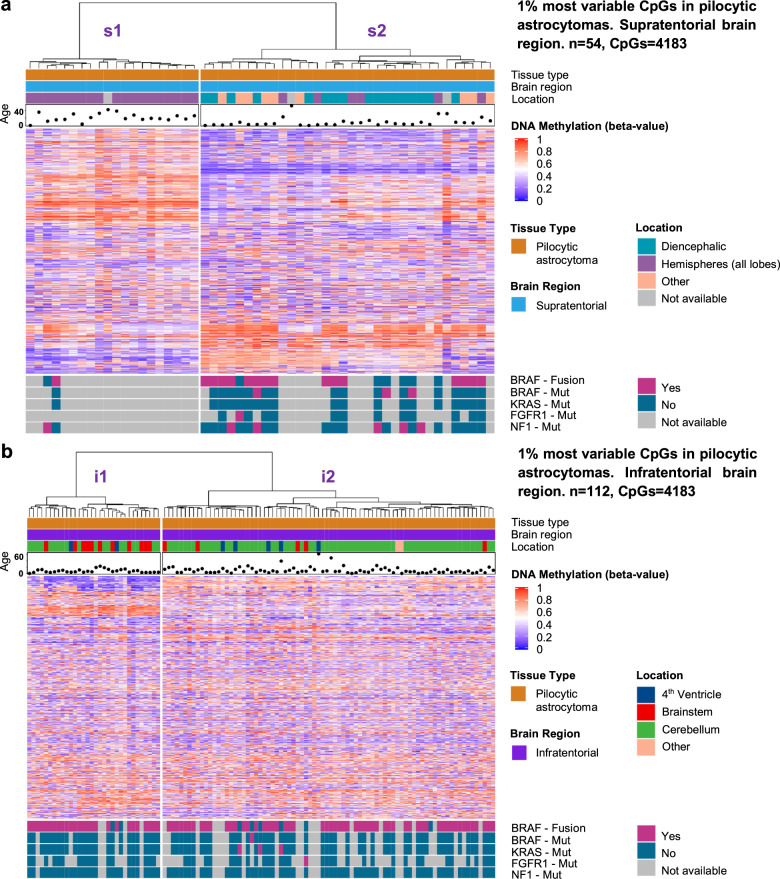
Hierarchical clustering of pilocytic astrocytomas. Hierarchical clustering incorporating the supratentorial or infratentorial PA cohort based on the tumor supratentorial (**a**) or infratentorial (**b**) cohort most variable one percent of CpGs (variability measure: standard deviation, distance measure: pearson, agglomeration: ward.D). The age of the patients in years, the exact tumor location as well as major genetic alterations are indicated. In the supratentorial brain, the s1 cluster is enriched for adult hemispheric PAs (**a**). In the infratentorial brain, in contrast, no age or location effect is detected in the clustering (**b**).

**Fig. 3:**
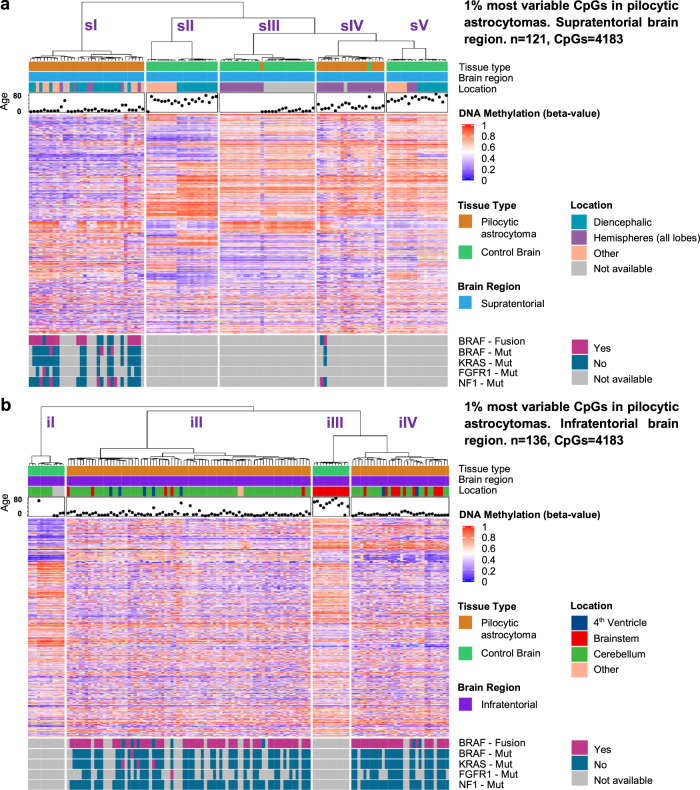
Hierarchical clustering of pilocytic astrocytomas and control brains. Hierarchical clustering incorporating the supratentorial or infratentorial PA cohort based on the tumor supratentorial (**a**) or infratentorial (**b**) cohort most variable one percent of CpGs (variability measure: standard deviation, distance measure: pearson, agglomeration: ward.D). The analysis shows a clear distinction between tumor and control brain tissue for both locations. In addition, the clustering indicates a distinction between PA clusters sI and sIV, enriched for pediatric PAs vs. adult hemispheric PAs, respectively (**a**). In the infratentorial brain, in contrast, pediatric and adult PAs cluster together, with different tumor locations in each subcluster (**b**).

The hierarchical clustering analysis showed two distinguishable groups among supratentorial PAs (Fig. 2a). Importantly, the first subcluster (cluster s1) included 17 adults out of 20 patients, whereas the second PA subcluster (cluster s2) only contained four adults out of 34 (*p* < 0.001, Fisher exact test). The median age of the patients in cluster s1 was 19 years, as compared to 4 years for cluster s2. When subdividing supratentorial PAs by exact tumor location, all PAs from cluster s1 (including a vast majority of adult PAs) appeared to be hemispheric PAs. In cluster s2 (including a vast majority of pediatric PAs), only six tumors, three pediatric and three adult PAs, were hemispheric. This finding points towards a potential location and age effect in supratentorial PAs. In contrast, we did not detect any age or location effect in infratentorial PAs, with pediatric and adult PAs clustering together, (Fig. 2b, median age of 6 and 8 years for the two clusters i1 and i2, respectively) and no specific tumor location dominating one given cluster. Including pediatric and adult normal brain tissues to the hierarchical clustering analyses confirmed the identified clusters, with the same segregation between PA groups and additional clusters containing normal brain tissues (Fig. 3). To validate the stability of the clusters obtained by the hierarchical cluster analysis, we calculated the bootstrap probability values, which correspond to the frequency of identifying a given cluster after bootstrap re-sampling. This analysis supported the stability of the clusters (Supplementary Fig. 1a), as did a clustering analysis performed using not only the 1% most variable CpGs, but the top 2 or 5% (Supplementary Fig. 1b–e). Visualization on t-distributed stochastic neighbor embedding (t-SNE) plots identified distinctly isolated clusters of normal brain tissues (Supplementary Fig. 2) for both supratentorial and infratentorial samples. Importantly, supratentorial PAs segregated into two main clusters on the t-SNE plots, clearly different in terms of proportions of adult patients. Infratentorial PAs of pediatric, adolescent and adult patients did not segregate on the tSNE plots. Even with a multivariate analysis taking into account the tumor location, we still detected an age-related difference among supratentorial PAs. Most importantly, the cluster sI comprising mostly pediatric PAs, revealed an average age difference of more than 10 years to the second supratentorial PA cluster sIV (*p* value < 0.04, see Supplementary Table 2). However, there is also a strong effect due to the exact tumor location within supratentorial PAs, with most of the hemispheric PAs of the cohort belonging to the adult PA cluster.

In addition to the hierarchical clustering based on the most variable CpGs within PAs, we also performed a hierarchical clustering analysis based on the most variable CpGs within all control and tumor samples (Supplementary Fig. 3). The sample assignment to specific clusters was nearly identical to the groups obtained based on the most variable CpGs between PAs only (see Figs. 2 and 3). For the supratentorial cohort, this analysis showed two distinct PA subclusters with marked differences in terms of patient age, supporting the age-related differences among supratentorial PAs. For the infratentorial cohort, PAs were clearly distinct from control samples, but adult and pediatric cases did not form separate clusters, confirming our findings related to the hierarchical clustering based on the most variable CpGs between PAs within the respective PA locations. Methylation profiles were independently useful regardless of driver mutations, as suggested by driver gene annotations on tSNE plots (Supplementary Fig. 4). Furthermore, supratentorial adult PAs still clustered separately after including additional low-grade gliomas to the analysis (e.g., pleomorphic xanthoastrocytomas, angiocentric gliomas, central neurocytomas, chordoid gliomas, desmoplastic infantile astrocytomas and gangliogliomas, oligodendrogliomas, subependymal giant cell astrocytomas) (Supplementary Fig. 5).

To test whether the age and location effects observed in supratentorial PAs might be due to different proportions of non-tumor cells present in the tumor tissue, we performed a bioinformatic inference to estimate the fractions of the major immune cell types in PAs based on our methylation cohort [19]. We did not detect any major difference in the inferred proportions of immune cell types between the different age groups (Supplementary Fig. 6), indicating that the age and location effect identified in supratentorial PAs is probably not directly influenced by immune infiltration. To further analyze the potential origin of the observed age and location-related clustering amongst supratentorial PAs, we validated the results with additional statistical methods, which confirmed the age and location effects (Supplementary Figs. 7 and 8). In addition, the copy-number profiles appeared as more similar across age groups for infratentorial PAs, as compared to supratentorial PAs (Supplementary Fig. 9, Supplementary Table 3).

Taken together, our analyses of the methylation patterns indicate differences between adult and pediatric PAs in the supratentorial brain, but no major age or location-related difference in the infratentorial brain.

**Fig. 4:**
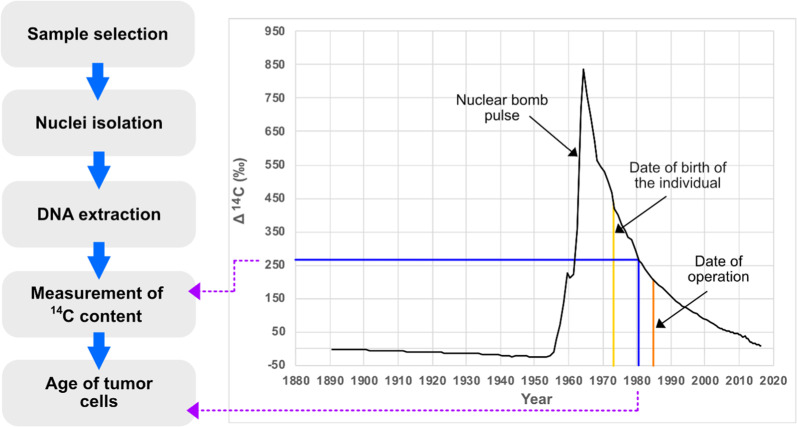
Workflow and strategy for retrospective birth-dating of tumor samples. After nuclei isolation from the tumor and control brain tissues, DNA is extracted and the ^14^C content of each sample is measured by accelerator mass spectrometry. The ^14^C content in each sample’s DNA provides a date mark to assess when the tumor was formed. The black curve shows the levels of ^14^C in the atmosphere, which have been stable over long time periods, with the exception of a large addition of ^14^C from 1955 to 1963 due to nuclear bomb tests. The date of birth of the individual and the date of the surgery are indicated by a yellow and an orange vertical line in the plot, respectively. The ^14^C concentration in genomic DNA from the cell population of interest is measured by accelerator mass spectrometry (shown by the horizontal blue line). The measured ^14^C value is related to the atmospheric levels (black curve) to establish at what time point the ^14^C value corresponded (indicated by the vertical blue line). The year is read off the *x*-axis, giving the birth date of the cell population. ^14^C levels from modern samples are by convention given in relation to a universal standard and corrected for radioactive decay, giving the Δ^14^C value.

To further test this hypothesis using an independent approach, we sought to determine the age of adult PA cells. We assessed the time point of the formation of adult PAs by measuring the concentration of nuclear bomb test-derived ^14^C in genomic DNA. When a cell is generated, it integrates ^14^C in the synthesized genomic DNA with a concentration corresponding to that in the atmosphere at that time, resulting in a date mark in the DNA (Fig. 4). We selected 14 tissue samples for retrospective birth-dating, including five adult and three pediatric PAs with confirmed molecular diagnosis and characterized by neuropathological analysis (Supplementary Fig. 10), two normal cerebellum samples (negative controls, no turnover) and four glioblastoma samples (positive controls, substantial turnover due to the high-proliferation rate). The characteristics of these tissues are summarized in Table 1, including the age of the individual at sample collection, the brain region from which the tissue was taken, the purity of the cells of interest, genetic features of the tumors and proliferation index. The majority of the analyzed PAs were located in the infratentorial brain, with 4/8 PAs in the cerebellum. The remaining samples were from less common locations for PAs, such as the pineal gland region (supratentorial brain), the diencephalic region (supratentorial brain) and the ventricles (third ventricle, supratentorial brain; fourth ventricle, infratentorial brain). All underlying genetic alterations detected in these PAs were previously reported (Table 1), including rare alterations, such as *KRAS* mutations [20].

**Table 1:** Characteristics of the tissue samples used for retrospective ^14^C dating.

Case	Date of birth	Diagnosis/tissue type	Age at surgery (years)	Brain region	Supratentorial or infratentorial	Purity (%)*	MAPK alteration	Proliferation index (%)**
1	1988	Pediatric pilocytic astrocytoma	9	Cerebellum	Infratentorial	90	KIAA1549:BRAF 16_9	2–3
2	1993		2	3rd ventricle	Supratentorial	80	KIAA1549:BRAF 16_9	8–10
3	1983		10	Cerebellum	Infratentorial	95	NA	NA
4	1968	Normal cerebellum	NA	Cerebellum	Infratentorial	95	Not applicable	<1
5	1950	Glioblastoma	59	NA	NA	NA	Not applicable	10–30
6	1970	Adult pilocytic astrocytoma	25	Cerebellum	Infratentorial	90	KIAA1549:BRAF 16_11	NA
7	1970		26	Cerebellum	Infratentorial	70	KIAA1549:BRAF 16_9	NA
8	1979		26	Diencephalic region	Supratentorial	100	KIAA1549:BRAF 16_9	1
9	1972		40	4th ventricle	Infratentorial	80	KRAS mutation	<1
10	1959		33	Pineal region	Supratentorial	80	MAPK alteration unknown	2
11	1974	Normal cerebellum	35	Cerebellum	Infratentorial	95	Not applicable	<1
12	1970	Glioblastoma	22	Hemispheres	Supratentorial	>85%	Not applicable	30
13	1970	Glioblastoma	22	Hemispheres	Supratentorial	90%	Not applicable	27
14	1988	Glioblastoma	6	Hemispheres	Supratentorial	90%	Not applicable	15

*NA* not available, *MAPK* mitogen-activated kinase.

*Tumor content was evaluated by pathological investigation of hematoxylin and eosin-stained samples.

**Proliferation index was determined as Ki67 labeling index.

**Fig. 5:**
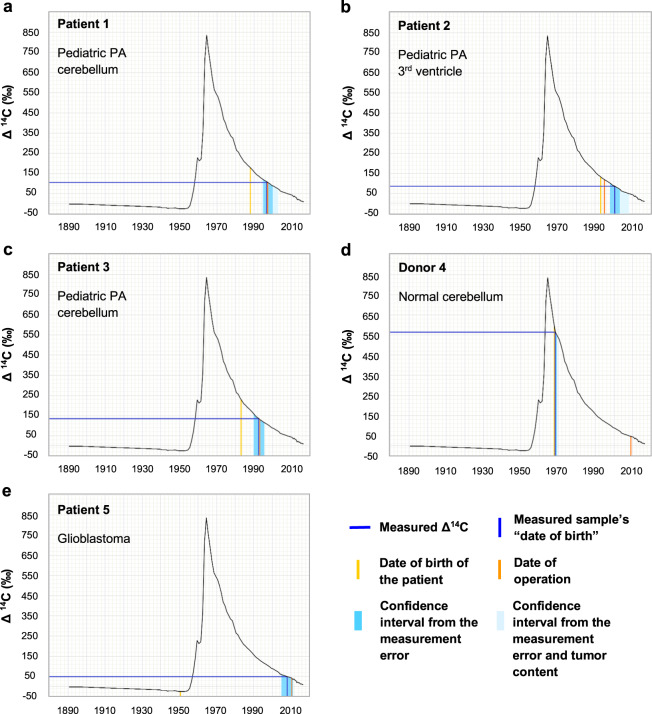
The results of retrospective ^14^C dating of control samples. The black curve shows the values of the ^14^C concentration in the atmosphere of the Northern hemisphere from 1890. Using this curve and the experimentally determined Δ^14^C for the given pediatric PA samples (**a**–**c**), **d** normal cerebellum, **e** glioblastoma, the date when the cells from the respective tissue sample were generated can be calculated (dark blue lines). In addition, for each donor (**a**–**e**), the date of birth of the individual (yellow) and the date of surgery (orange, identical to the date of sample collection) are indicated. Further details on these samples can be found in Table 1: **a**–**e** correspond to sample IDs 1–5, respectively. The confidence intervals reflect the accelerator mass spectrometry measurement error of the sample’s “date of birth” (blue shading) and the worst-case scenario including possible contaminations by non-tumor cells (non-neuronal cells from the cerebellum in **d**) in addition to the measurement error (light blue shading). ^14^C levels from modern samples are by convention given in relation to a universal standard and corrected for radioactive decay, giving the Δ^14^C value.

We assessed the average date of generation of the cells in each sample and calculated two types of confidence intervals for each (Fig. 5). First, we calculated the confidence intervals reflecting the measurement error of the accelerator mass spectrometry analysis (shown as dark blue shading around each sample’s “date of birth”). Second, we simulated the worst-case scenarios considering, in addition to the measurement error, possible contaminations of the tissue, such as non-tumor cells, which could be as old as the individual or contemporary cells generated right before sample collection, in the two extreme scenarios (shown as light blue shading).

For pediatric PAs, the calculated date of birth of the tumor cells was close to the date of surgery, as expected due to the short time span between the birth of the individual and the sample collection (Fig. 5a–c). For pediatric PA patient 2, the relatively recent sample collection combined with a very short time span of 2 years between the date of birth of the individual and the sample collection reached a technical limit of the method. As outlined previously, the accuracy of individual datings is about ±2 years, with variations depending on the date of the sample collection and the date of birth of the individual [11]. For pediatric patients 1 and 3, however, with a larger time span between the birth of the individuals and the sample collection, the calculated date of generation of the tumor cells was concordant with the date of surgery. As expected, the average age of the cells from the normal cerebellum tissues used as negative controls matched to the age of the individuals, due to the low turnover in cerebellar neurons after birth (Fig. 5d and Supplementary Fig. 11). In the adult glioblastoma sample used as a positive control, as expected for a tumor with a high proliferation index, the average age of the cells was only 3 years younger than the time of the sample collection. In line with this, analyses of the evolutionary trajectory of adult glioblastomas showed that the tumor initiation likely starts two to seven years before diagnosis [21]. In the pediatric glioblastoma samples used as positive controls, the average age of the cells was very close to the time of the sample collection, in line with pediatric tumors with a high proliferation index (Supplementary Fig. 11). Taken together, these results show that we can apply this approach to assess the age of adult PAs.

**Fig. 6:**
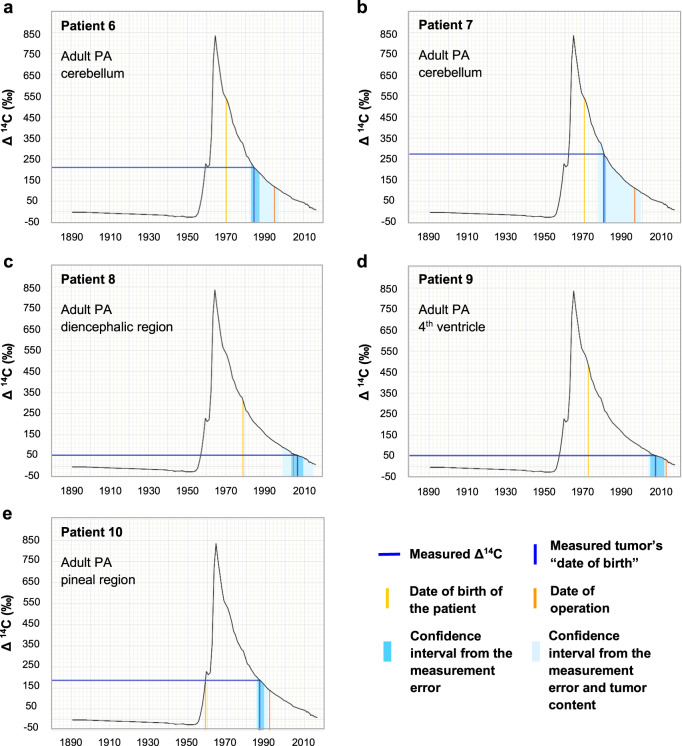
The results of retrospective ^14^C dating of adult pilocytic astrocytoma (PA) samples. The black curve shows the values of the ^14^C concentration in the atmosphere of the Northern hemisphere from 1890. Using this curve and the experimentally determined Δ^14^C for the given adult PA samples (**a**–**e**), the date when the cells from the respective tissue sample were generated can be calculated (dark blue lines). In addition, for each patient (**a**–**e**), the date of birth of the individual (yellow) and the date of surgery (orange, identical to the date of sample collection) are indicated. Further details on these patients can be found in Table 1: **a**–**e** correspond to PA IDs 6-10, respectively. The confidence intervals reflect the accelerator mass spectrometry measurement error of the tumor’s “date of birth” (blue shading) and the worst-case scenario including possible contaminations by non-tumor cells in addition to the measurement error (light blue shading). ^14^C levels from modern samples are by convention given in relation to a universal standard and corrected for radioactive decay, giving the Δ^14^C value.

Next, we showed that a subset of adult PAs originated at least 10–20 years before diagnosis (Fig. 6a, b). The average age of the tumor cells in these patients was far from the date of surgery, and a subset of these tumor cells must have been much older than this average age. The tumor likely developed during childhood already, and not from a precursor cell present in the adult brain. In contrast, for other adult PAs, the average age of the tumor cells was very close to the date of diagnosis (Fig. 6c–e). For patient 10, who was born on the upward slope of the bomb pulse, the date of generation of the tumor cells could theoretically be matched to the bomb peak in two places. Even though this case cannot be resolved without ambiguity, since pineal PAs generally lead to early symptoms [22], we believe it to be unlikely that the tumor may have developed early in life and remained undetected for three decades. In general, the time span from tumor development to symptom detection and diagnosis may be linked with the tumor location, with tumors developing close to the midline possibly expected to generate symptoms earlier.

Interestingly, adult PAs dated as “old tumors” developed in the cerebellum (infratentorial brain), whereas adult PAs dated close to the date of surgery developed in other brain regions. Even though we cannot generalize due to the size of the cohort, this distinction identified by retrospective birth-dating supports the age and location effects observed in the DNA methylation patterns of supratentorial PAs but not for infratentorial PAs. Taken together, this is the first evidence showing that at least a subset of adult PAs develops early in life. Our data suggest that adult and pediatric PAs developing in the cerebellum are closely related and potentially develop from early precursor cells. Supratentorial PAs might show age and location-specific differences.

## Discussion

Knowing when a tumor started developing has major clinical implications in terms of surveillance and possibilities for early therapeutic intervention. Patients with tumors detected at a small size have a significantly better prognosis in a number of tumor entities. Therefore, different strategies have been applied to assess when tumors originate, including sequencing-based approaches [21, 23], retrospective birth-dating [17] and clinically implemented screening programs for cancer-prone individuals [24].

The supportive evidence gained from the analysis of methylation patterns in 257 PA and control brain samples provides important complementary insights to our retrospective birth-dating study. The size of the cohort and challenges linked with the availability of adult PA tissue for carbon-dating limit the generalization of our findings. The requirements for large amounts of fresh-frozen tissue (at least 300 mg) with a high tumor content and within specific ranges for the dates of birth of the patients and of sample collection make it impossible to assemble a large dataset for retrospective birth-dating measurements. However, combining retrospective birth-dating for ten cases with analyses of DNA methylation patterns reflecting the cell of origin for 257 cases revealed (1) an early origin for infratentorial PAs (with evidence for a subset of adult cerebellar PAs being formed several decades before diagnosis) (2) marked age and location-related differences in supratentorial PAs.

The large inter-individual variability, possibly due to the different brain regions where these tumors develop and distinct oncogenic lesions, represents a challenging factor. Supratentorial and infratentorial PAs occur with different frequencies in children and adults and are linked with distinct genetic alterations and gene expression signatures [25]. Furthermore, the location of the PAs likely affects the time span between the tumor development and the time of symptom appearance, surgery and sample collection. For instance, the cerebellum is more capable of coping with structural impairment as compared to other brain regions, with the term “cerebellar reserve” referring to the unique capacity of this brain region to compensate for tissue damage or loss of function [26]. Therefore, it is expected that the “date of birth” of PA cells shows a wide variation among patients.

Non-tumor cells present in the analyzed tissue also contribute to the measured ^14^C content and thereby to the calculated age of the cells. To maximize the precision of our ^14^C analyses, we selected PAs with a high tumor content (85% on average, see Table 1) and used the percentage of non-tumor cells assessed for each sample to correct for the contamination. In addition, we added an error margin to the measured tumor content to consider variations due to intra-tumor heterogeneity. We calculated confidence intervals considering that the percentage of non-tumor cells could be either very old cells (as old as the individual) or contemporary cells (matching to the date of sample collection). These worst-case scenarios are unlikely, as the non-tumor cells are mixtures of different cell types with variable turnover rates, including neurons, immune cells, astrocytes and oligodendrocytes. However, this allows defining confidence intervals, in addition to the confidence intervals from the measurement error of the accelerator mass spectrometry.

Methylation is a source of carbon exchange in the genome, which could potentially affect the retrospective birth-dating results. Nevertheless, even if all methyl groups were contemporary, addition of one carbon atom by methylation would be negligible, as calculated in a previous study [11].

Our results raise questions about the putative cell of origin of PAs in adults and children. Pediatric PAs have been hypothesized to originate *in utero*, with cerebellar PAs mirroring the transcriptomes of specific embryonic cerebellar cell clusters [27]. Even though our approach cannot assess whether the precursor cells giving rise to pediatric PAs still exist in adults, we provide the first evidence that at least a subset of adult PAs develops early in life. This early origin raises the question of the potential role of oncogene-induced senescence [28], the putative contribution of immune cells to keep these PAs under control for several decades, the role of hormones, inflammatory stimuli and other components of the microenvironment, and which events trigger the increase in tumor growth at a given time point. Longitudinal imaging data would be helpful for better understanding the growth kinetics of adult PAs. Taken together, our data suggest that adult and pediatric PAs developing in the infratentorial brain are closely related and potentially develop from early precursor cells, whereas supratentorial PAs might show age and location-specific differences.

## Material and methods

### Patient selection

For the retrospective ^14^C dating, fresh-frozen tissue samples were used. They were obtained from 14 donors: eight patients with histological diagnosis “pilocytic astrocytoma, WHO grade I” and methylome signatures corresponding to the molecular diagnosis “pilocytic astrocytoma”, four patients with histological diagnosis “glioblastoma, WHO grade IV”, and two donors without brain tumor admitted for autopsy (normal cerebellum tissue). The specimens were collected at the Cambridge Hospital, the Uppsala University, the Burdenko Neurosurgical Institute in Moscow and the Karolinska Institute in Stockholm. Informed consent was obtained from all patients or their relatives according to the respective institutional guidelines. All details related to this cohort are given in Table 1 and Supplementary Table 4.

Tissue samples used for the methylation profiling (156 PA and 72 control brain samples) were obtained from Gene Expression Omnibus accession number GSE109381 and processed as described previously [6]. Additional 19 control tissue samples were retrieved from the Allen Brain Atlas [29] and processed as outlined previously [8]. Furthermore, ten new adult PA samples were subject to DNA methylation profiling.

### Histopathological analysis

To confirm the diagnosis and assess the tumor cell content and the proliferative index of PAs used for the nuclei isolation and consequent retrospective ^14^C dating, tumor tissues were subjected to neuropathological review. Hematoxylin and eosin (H&E) staining and immunohistochemistry analyses were performed on 10 μm formalin-fixed tissue sections. For Ki67 stains, slides were washed with PBS for 5 min and then blocked with blocking solution (10% goat serum diluted in PBS, 0.2% Triton X-100) for 1 h. Slides were then incubated with the primary antibody, Ki67 (Abcam, 15580), at 1:100 dilution overnight at 4 °C and then visualized using the matching secondary antibody. H&E staining and Ki67 staining were evaluated by a neuropathologist.

### DNA methylation analysis

DNA was analyzed using the Illumina HumanMethylation450 BeadChip (450 k) as well as Infinium Methylation EPIC (850 k) array, as previously described [6]. CpG position overlapping with single nucleotide polymorphisms (SNP, version dbSNP 151) were excluded. To concatenate 450 k and 850 k array data, only CpGs covered by both technologies were kept. In addition, only CpGs located on autosomes were subject to analysis. Unsupervised hierarchical clustering was based on the most variable one percent of CpGs across the dataset (whole cohort or tumor cohort) as measured by standard deviation. Samples were clustered using 1-Pearson correlation coefficient as the distance measure and ward.D agglomeration. t-SNE was performed on the same dataset as unsupervised hierarchical clustering (R-package Rtsne v. 0.15, Van Der Maaten L and Hinton G. Visualizing data using t-SNE). PA clustering in relation to other low-grade gliomas was analyzed using data provided by Capper et al. [6]. Information on methylation-based DNA copy-number-variation was provided by the DKFZ internal analysis pipeline of the *Molecular Neuropathology* program (www.molecularneuropathology.org/mnp). Plots displaying frequencies of DNA copy-number variations were generated using the R-package *GenVisR* v. 1.22.0.

### Accelerator mass spectrometry

All accelerator mass spectrometry analyses were performed blind to identity of the samples. The DNA samples, suspended in DNase/RNase-free water (GIBCO/Invitrogen), were transferred to quartz combustion tubes and dried in a lyophilizer at 10^−3^ mbar. Excess CuO powder was added to each sample and the tube was evacuated and subsequently sealed using a high-temperature torch and placed in a furnace at 900 °C for 3.5 h to combust all carbon into CO_2_. The generated gas was purified and the CO_2_ cryogenically transferred into a miniature graphitization reactor (<1 mL) in the presence of zinc powder for the reduction process and iron powder as catalyst. The reactors were individually heated to 550 °C for 6 h where the conversion to graphite is completed. The samples were then measured at the Department of Physics and Astronomy, Ion Physics, of Uppsala University as previously reported. Strict laboratory routines for minimizing the introduction of background carbon are implemented which include prebaking of quartz and borosilicate tubes as well as iron, zinc and CuO powders. Large CO_2_ samples (>100 μg) were split, and δ^13^C was measured by stable isotope ratio mass spectrometry.

## Supplementary information

Supplementary Figures

Supplementary Table 1

Supplementary Table 2

Supplementary Table 3

Supplementary Table 4

Supplementary Methods
