# Supplementary material for: The age of adult pilocytic astrocytoma cells
Source: Oncogene. 2021 Mar 17;40(16):2830–41. doi: 10.1038/s41388-021-01738-0 (PMC8062266; doi:10.1038/s41388-021-01738-0)
Supplement: Supplementary file 1 — Supplementary Figures [file 41388_2021_1738_MOESM1_ESM.pdf]

Supplementary Figure 1

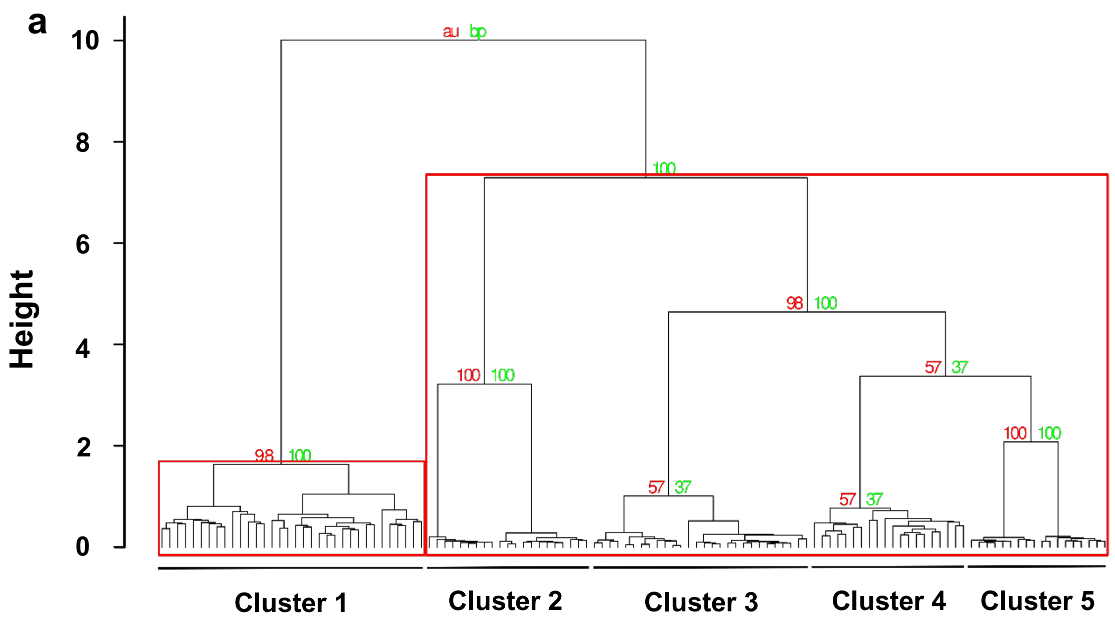

Supplementary Figure 1 (a). Cluster stability analysis via 1000-fold bootstrap resampling. Samples of the cluster with most adult pilocytic astrocytomas (cluster 4) showed to cluster stably (indicated by red boxes) after random sub-sampling.

bp, bootstrap probability values (shown in green)

Supplementary Figure 1

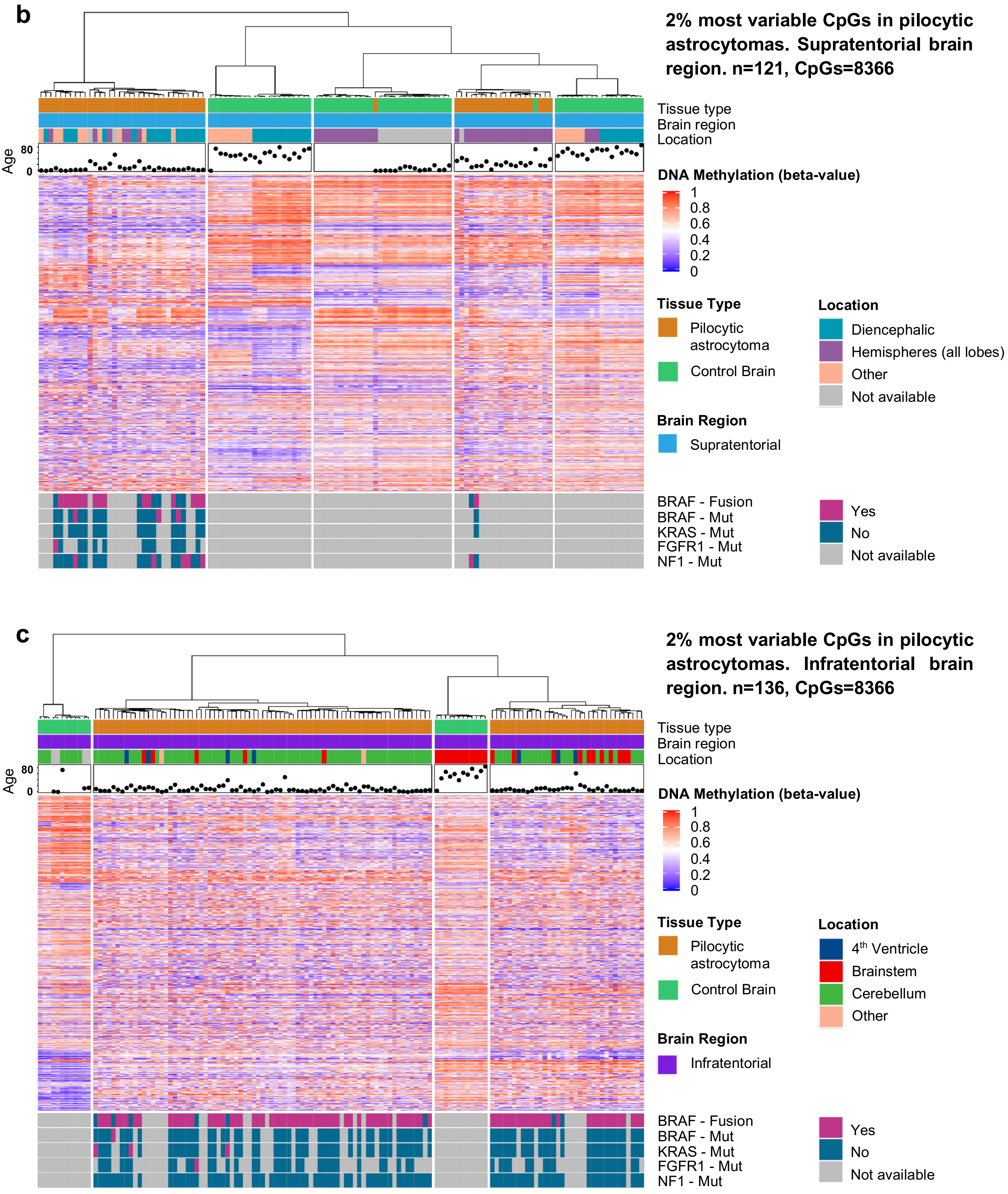

Supplementary Figure 1 (b-c). Hierarchical clustering incorporating the (b) supratentorial or (c) infratentorial PA cohort based on the tumor supratentorial (b) or infratentorial (c) cohort most variable two percent of CpGs (variability measure: standard deviation, distance measure: pearson, agglomeration: ward.D).

Supplementary Figure 1

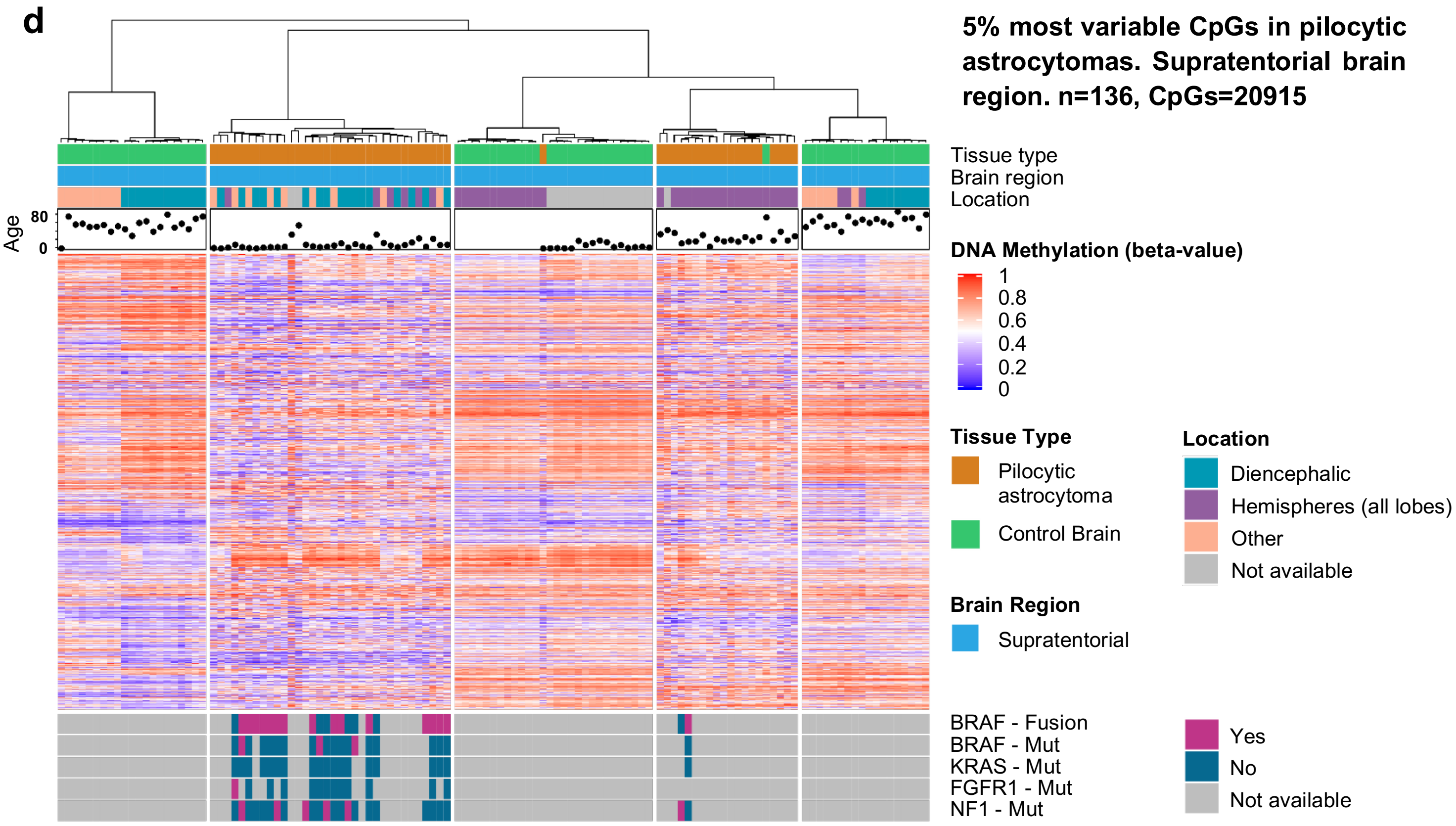

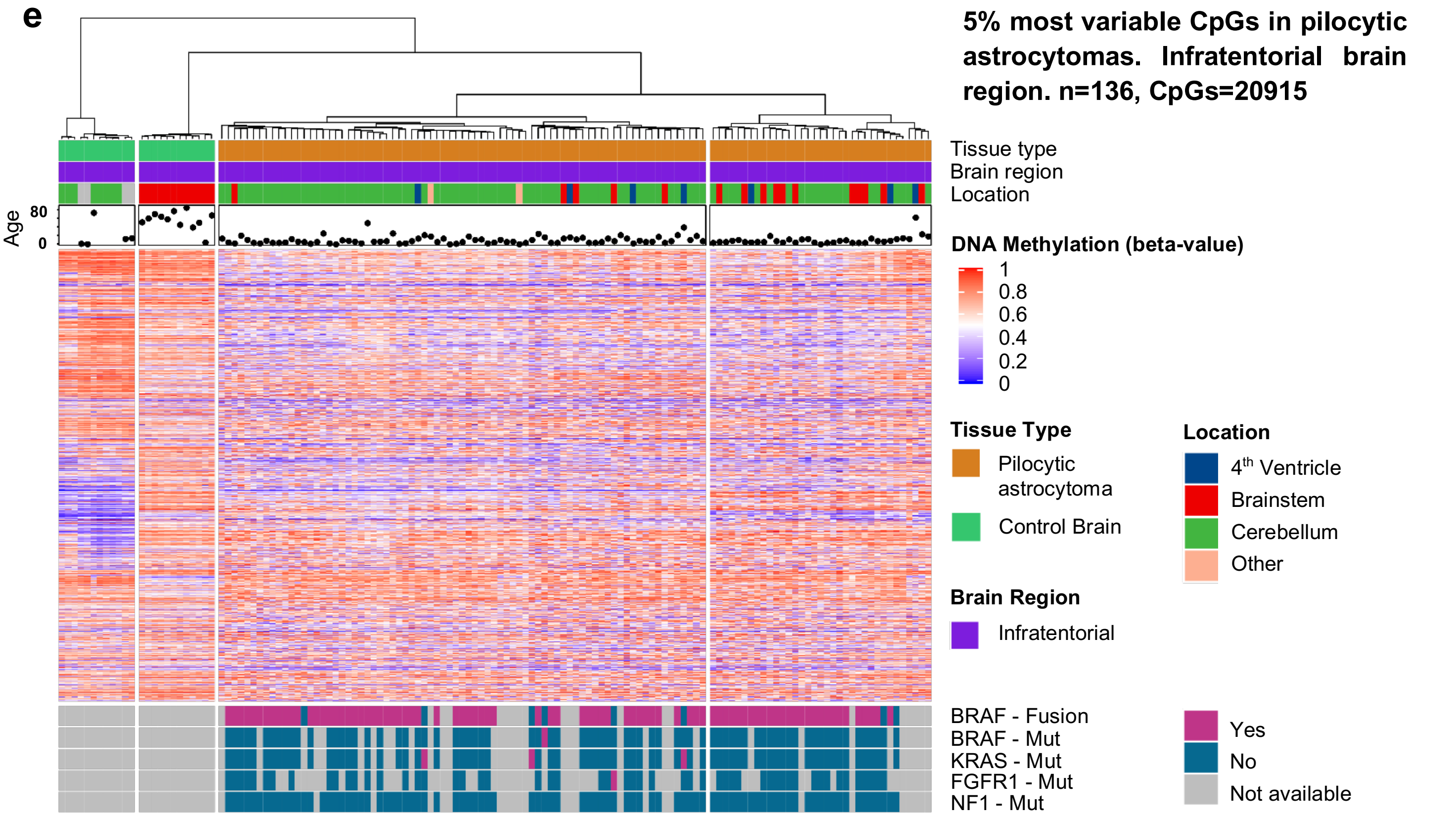

Supplementary Figure 1 (d-e). Hierarchical clustering incorporating the (d) supratentorial or (e) infratentorial PA cohort based on the tumor supratentorial (d) or infratentorial (e) cohort most variable five percent of CpGs (variability measure: standard deviation, distance measure: pearson, agglomeration: ward.D).

Supplementary Figure 2

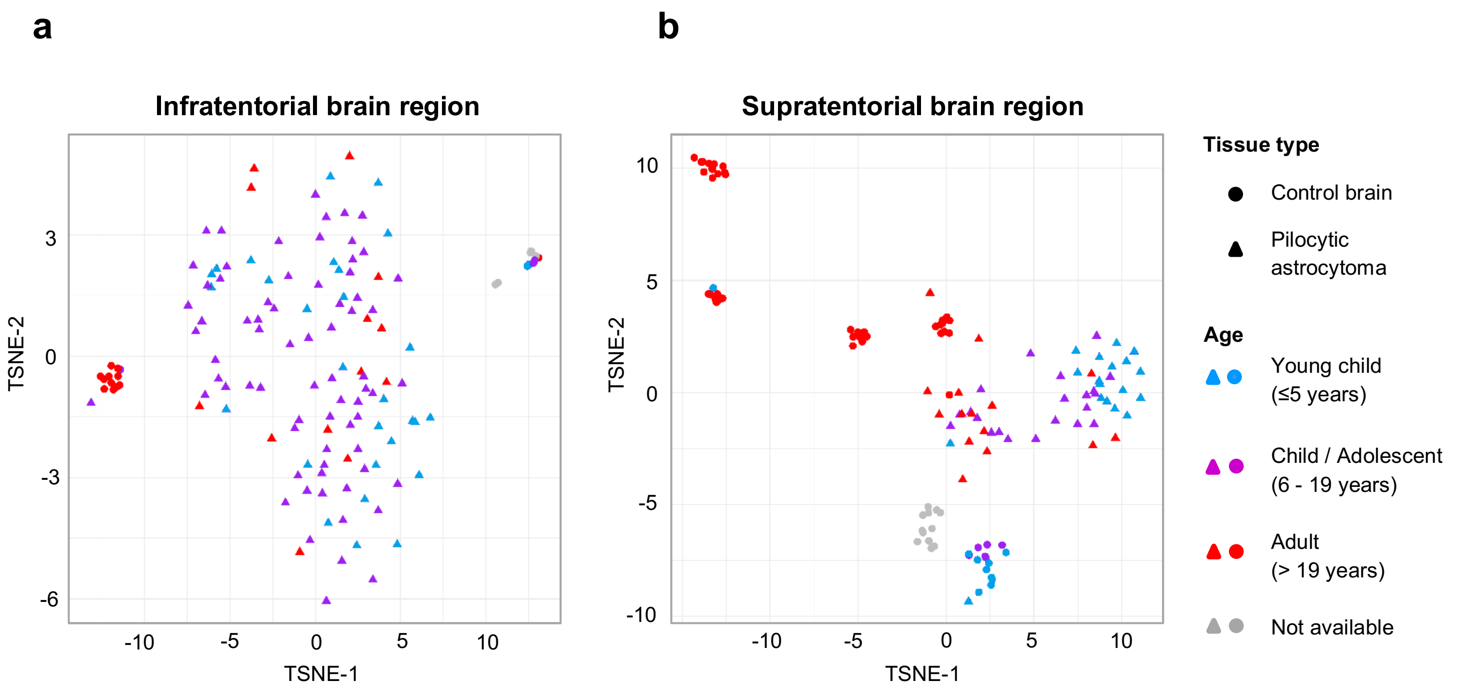

Supplementary Figure 2. T-SNE analysis for infratentorial and supratentorial PA and control brain samples. T-distributed stochastic neighbor embedding (T-SNE) applied to the 1% most variable tumor CpGs (measured by standard deviation) stratified by brain compartment. **a.** PA cases in the infratentorial brain region show no age-related separation. **b.** Supratentorial PAs show two distinct age-related groups, enriched for young children or adult cases, respectively.

Supplementary Figure 3

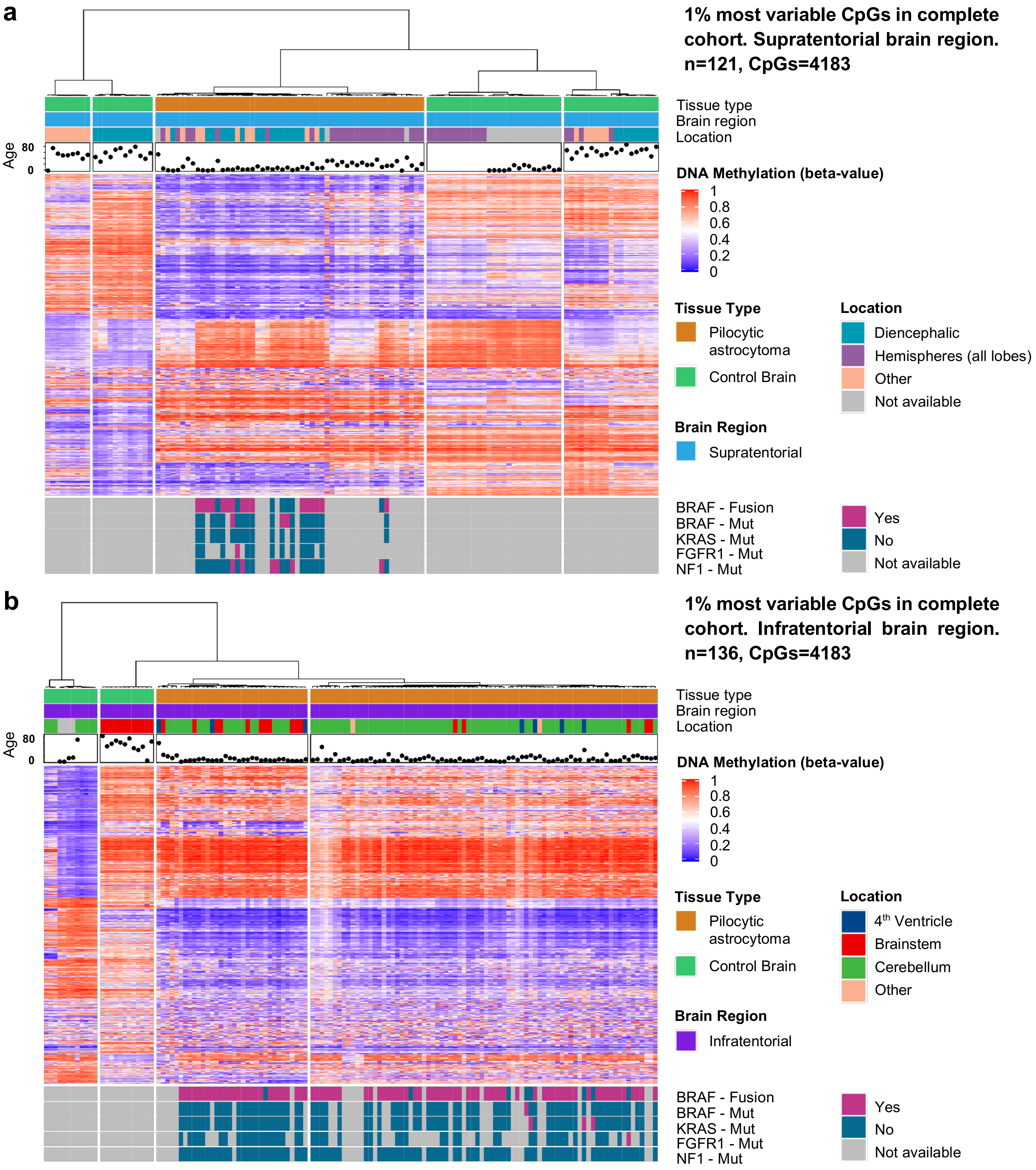

Supplementary Figure 3. Hierarchical clustering incorporating the whole **a.** supratentorial or **b.** infratentorial cohort based on the whole supratentorial (a.) or infratentorial (b.) cohort most variable one percent of CpGs (variability measure: standard deviation, distance measure: pearson, agglomeration: ward.D).

Supplementary Figure 4

Infratentorial pilocytic astrocytomas

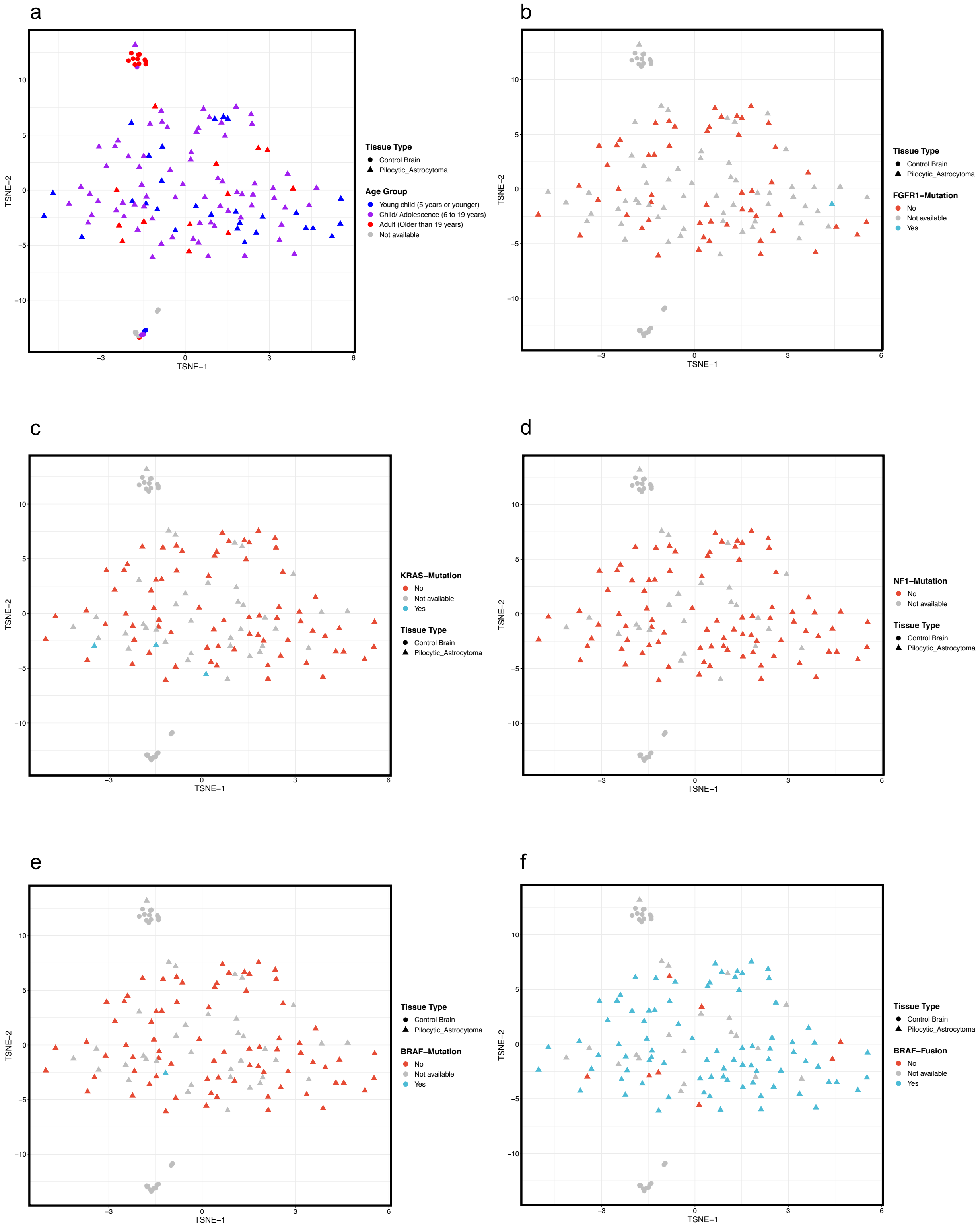

Supratentorial pilocytic astrocytomas

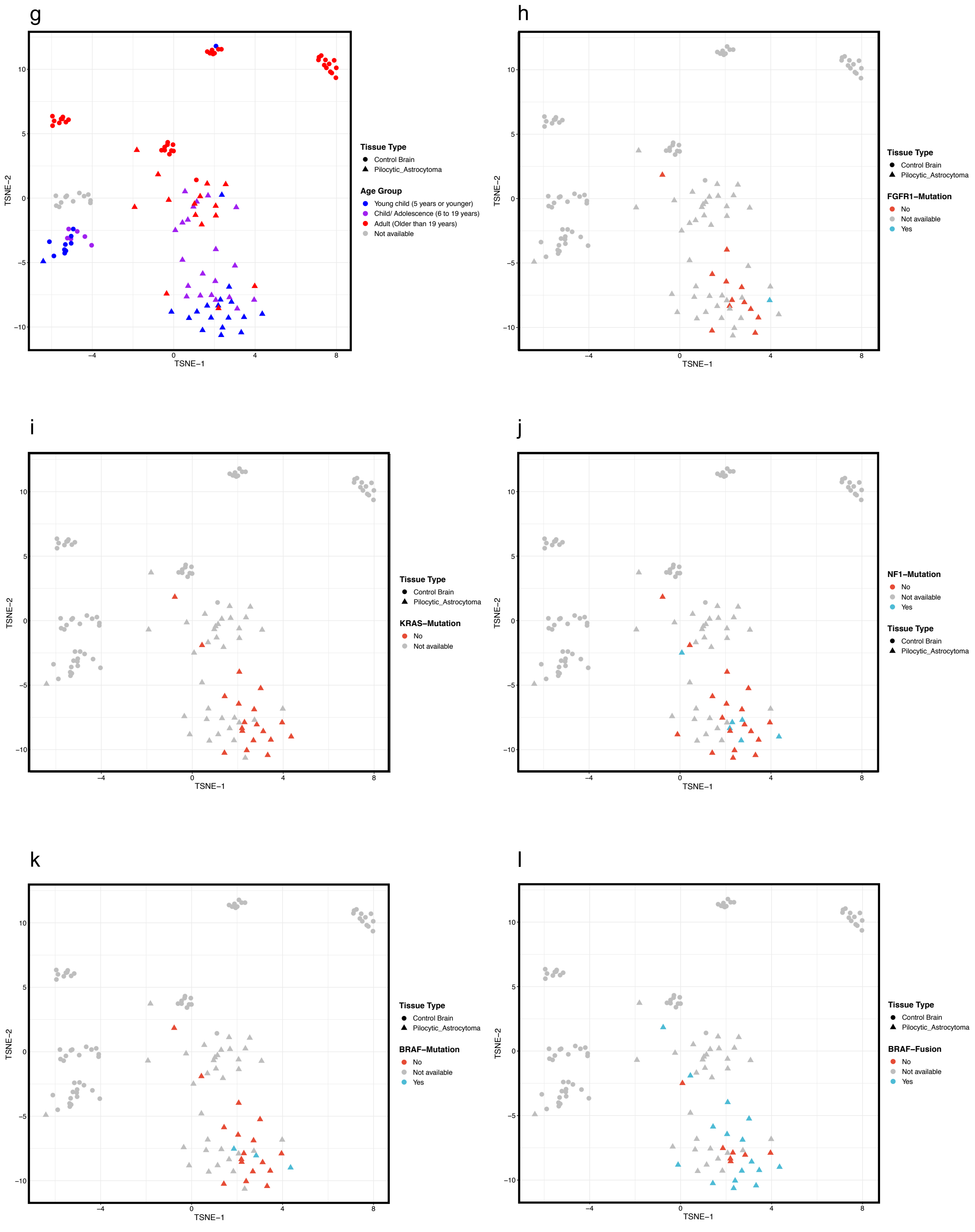

Supplementary Figure 4. Annotations of driver mutations on tSNE plots for infratentorial and supratentorial pilocytic astrocytomas. tSNE dimension reduction based on the most variable 1% CpGs (according to standard deviation) does not indicate the presence of a biologically different sub-group defined by mutational status within the infratentorial tissue cohort.

Supplementary Figure 5

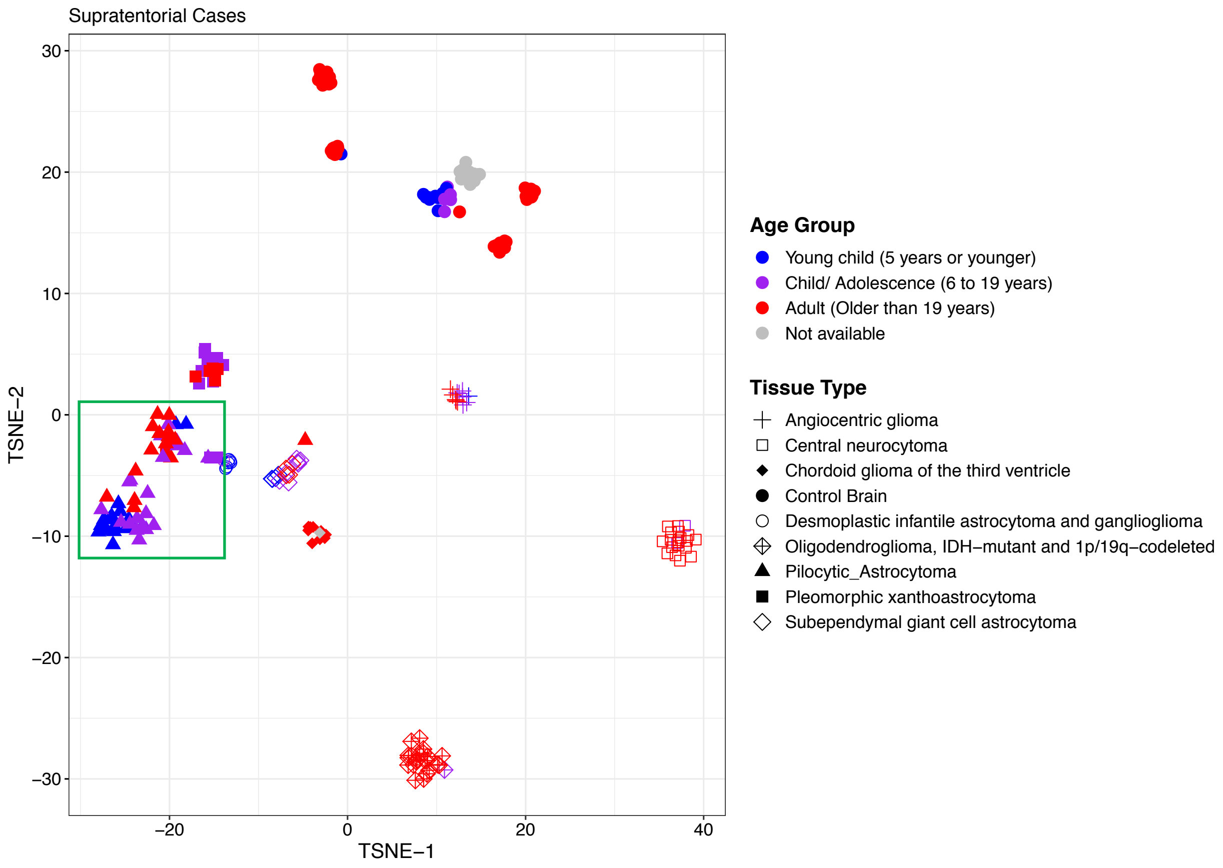

Supplementary Figure 5. tSNE plot for supratentorial tumors including low-grade gliomas from different tumor entities in addition to pilocytic astrocytomas. Green square highlights pilocytic astrocytomas. tSNE dimension reduction is based on the most variable 1% CpGs (according to standard deviation), stratified by brain compartment. The clusters mostly reflect the different tumor entities. Among supratentotrial pilocytic astrocytomas, two age-related subgroups are detectable.

Supplementary Figure 6

Immune cell composition in infratentorial and supratentorial pilocytic astrocytomas

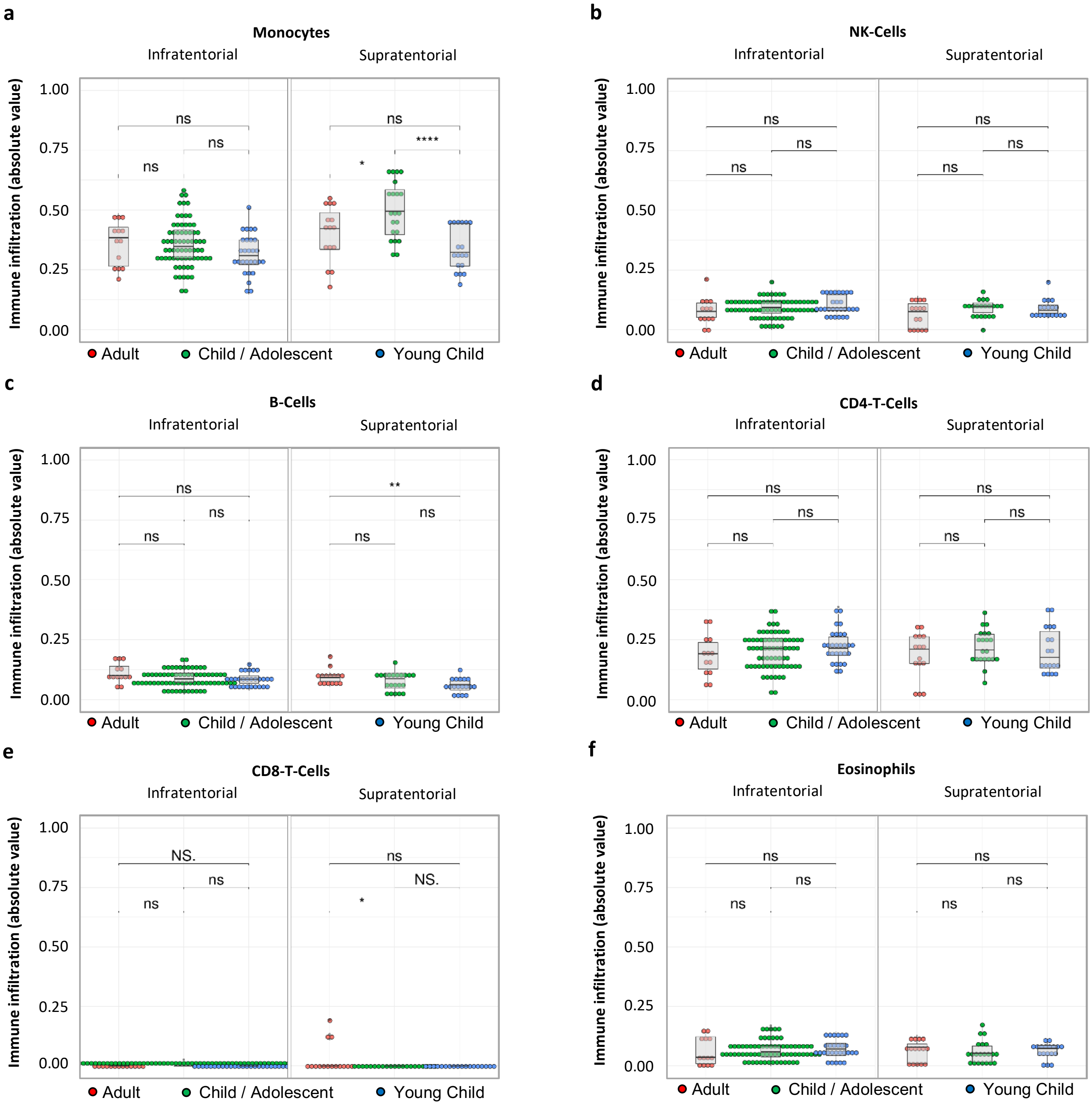

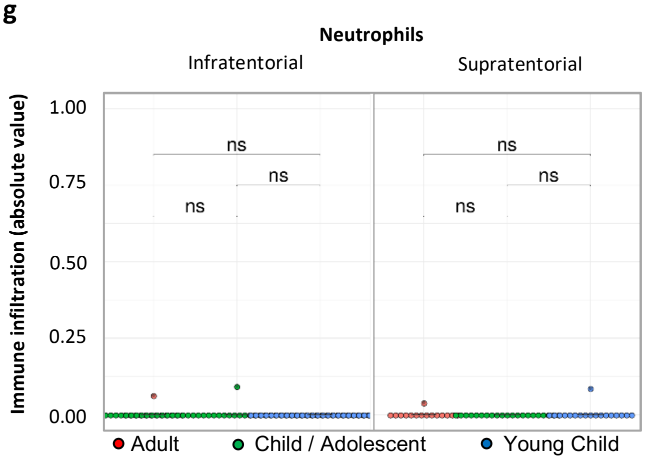

Supplementary Figure 6. In silico immune infiltration analysis of the PA cohort, based on DNA methylation, did not show any major difference in the abundance of immune cells between age groups (Wilcoxon test, $p <= 0.05$, arbitrary immune infiltration score, comparable between samples).

ns – not significant
NS – not tested due to the zero variance of the data

Adult: older than 19 years
Child / Adolescent: 6 to 19 years
Young child: 5 years or younger

Supplementary Figure 7

a

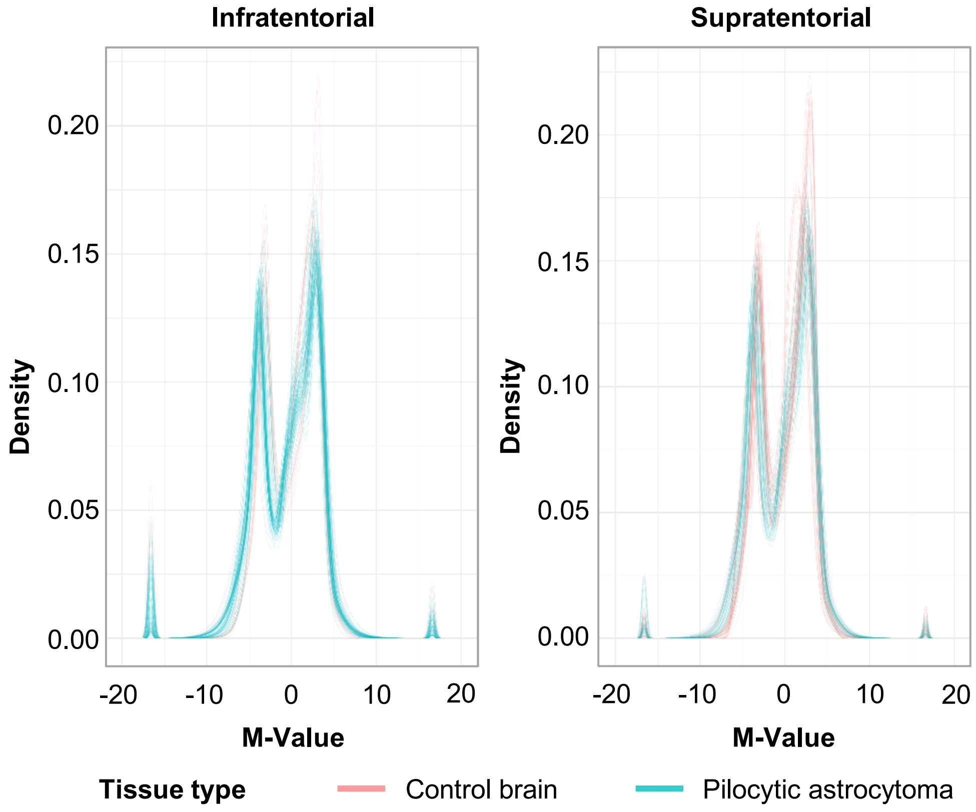

Supplementary Figure 7. M-value distribution and the results of the gaussian mixture model. **a.** m-value distribution of infra- and supratentorial samples show a multimodal distribution. **b.** Gaussian mixture model with age and immune infiltration scores of selected immune cells as independent variables. This model is able to reconstruct the initial m value distribution, with a zero centered asymmetrical residual distribution indicating no systematic error.

b

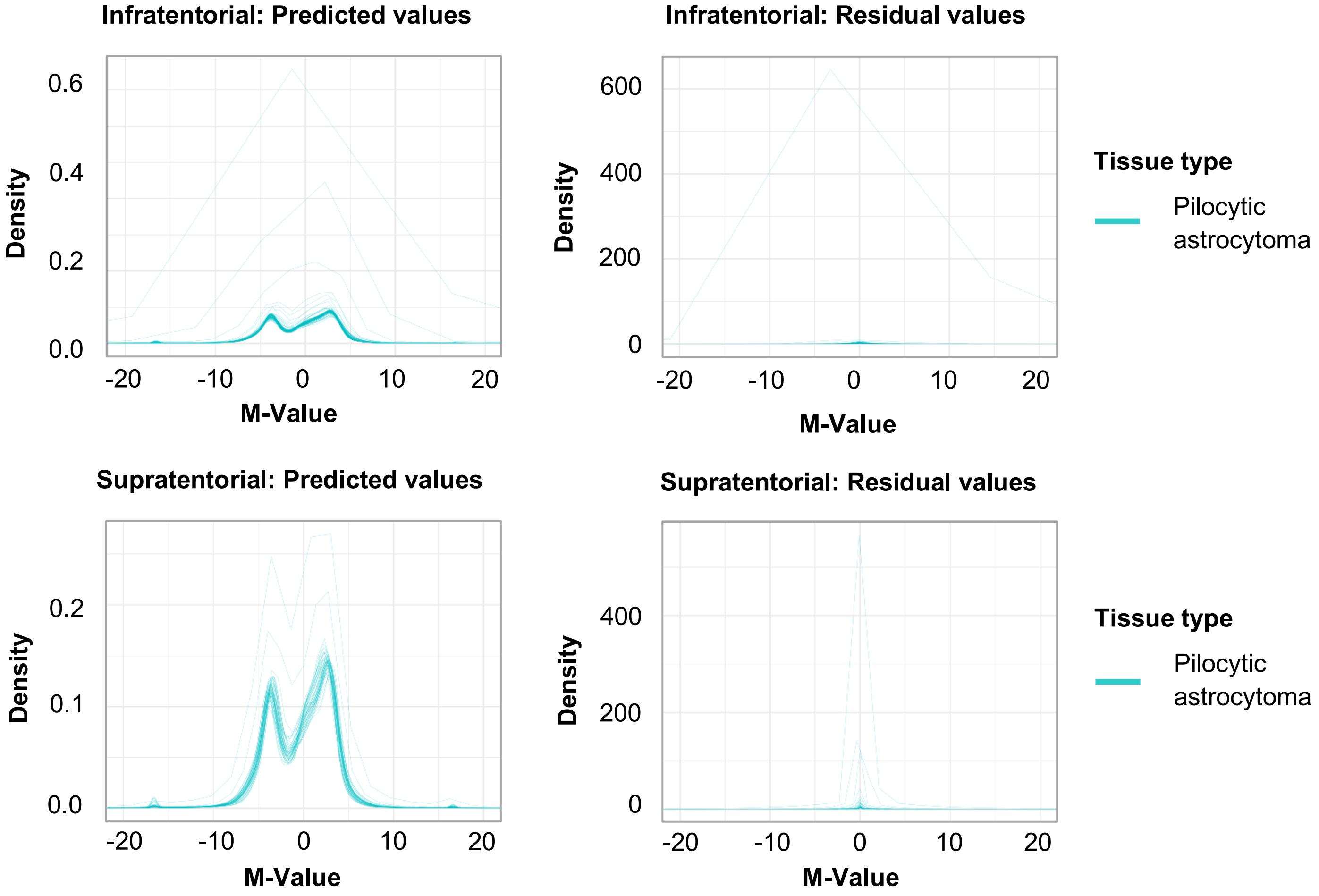

Supplementary Figure 8

a

1% most variable CpGs in pilocytic astrocytomas. Supratentorial brain region. n=54, CpGs=4190. Fitted values

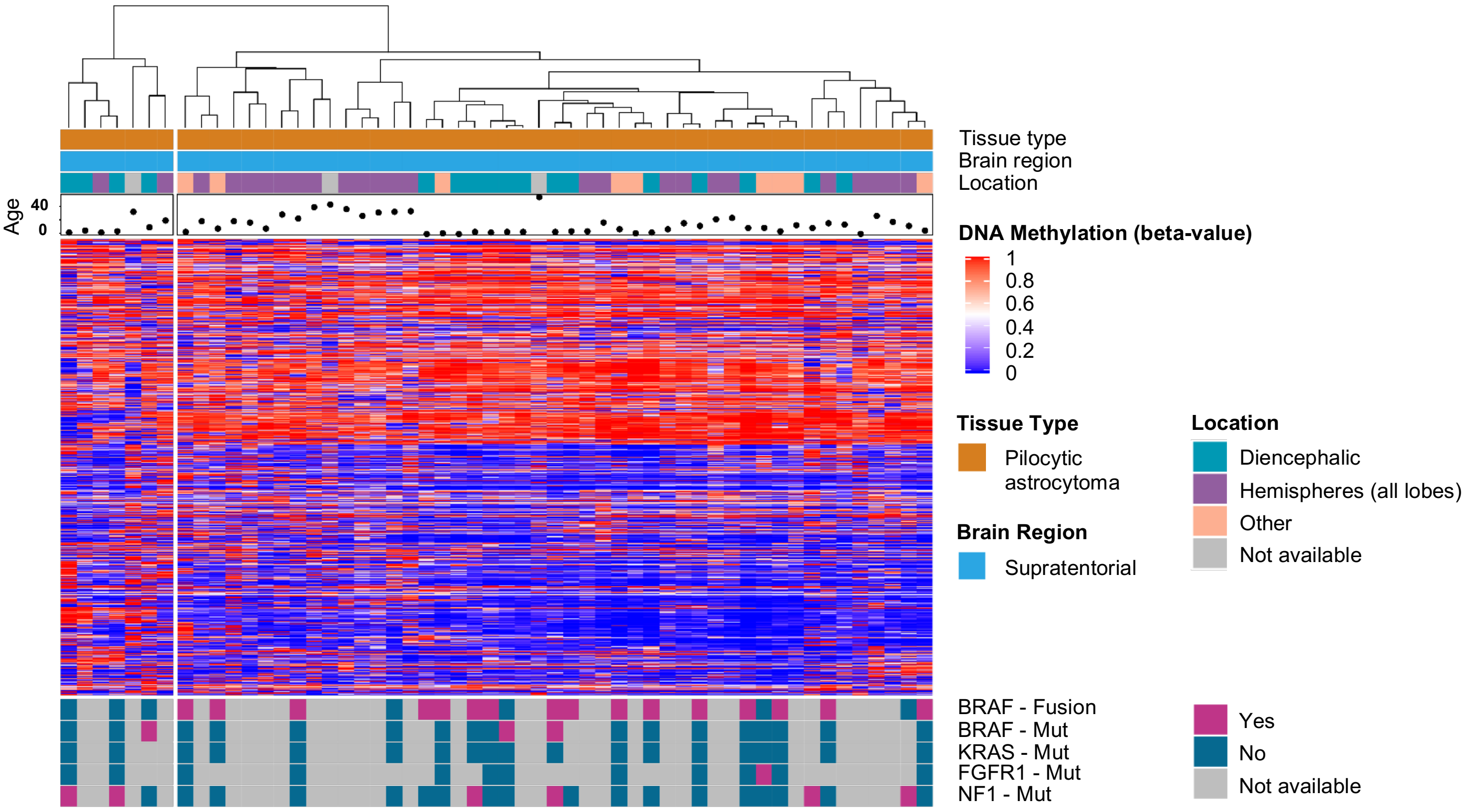

b

1% most variable CpGs in pilocytic astrocytomas. Supratentorial brain region. n=54, CpGs=4190. Residual values

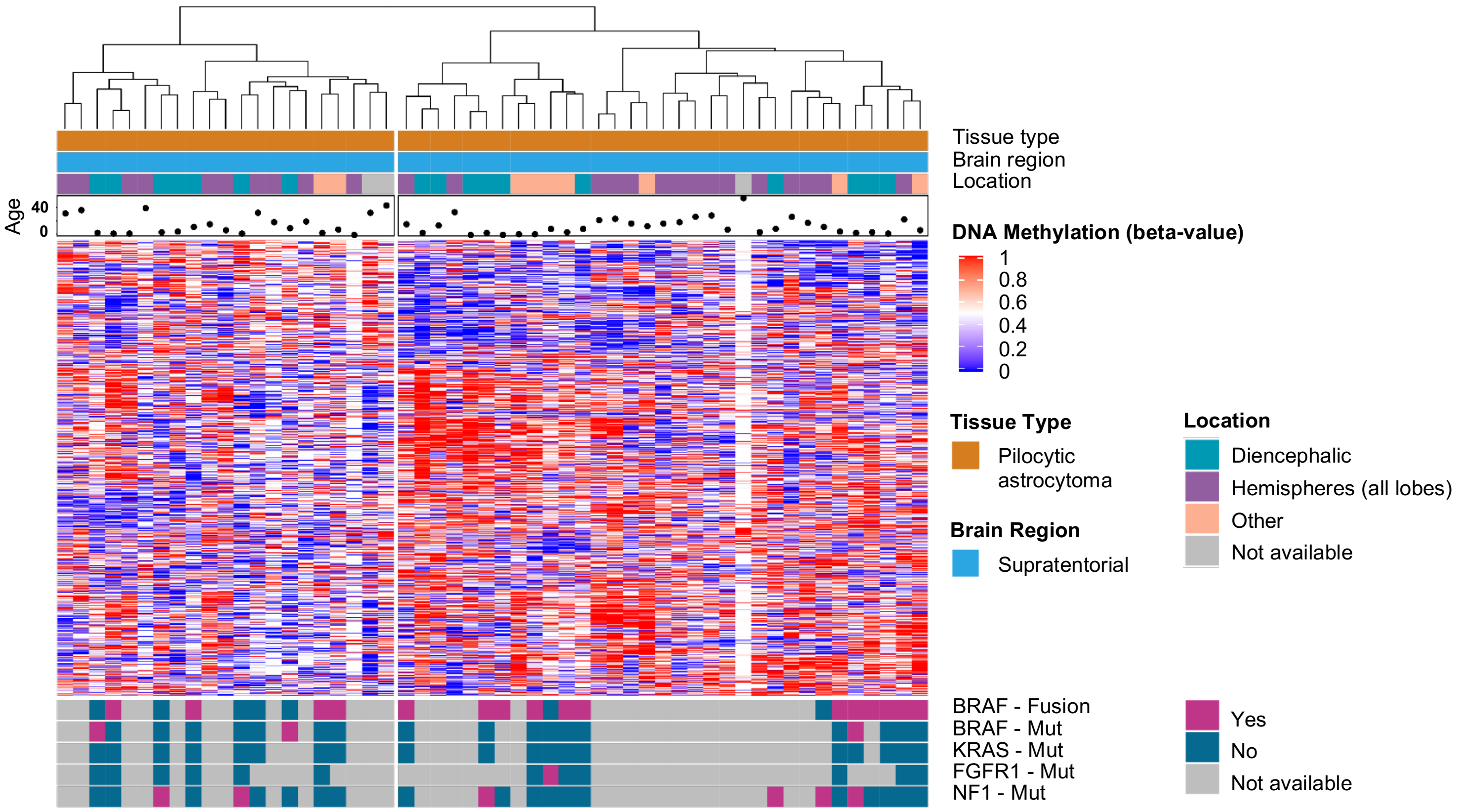

Supplementary Figure 8

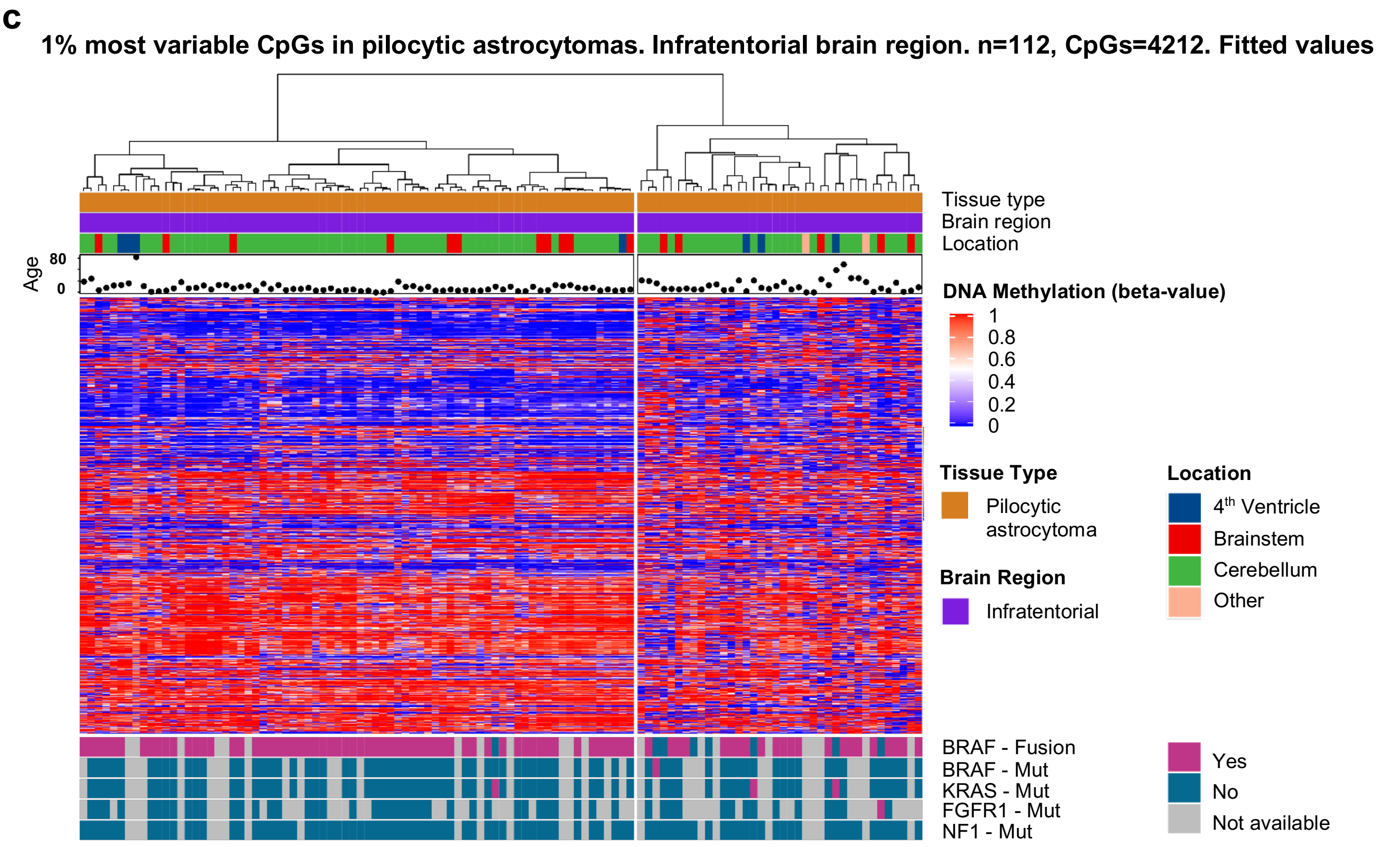

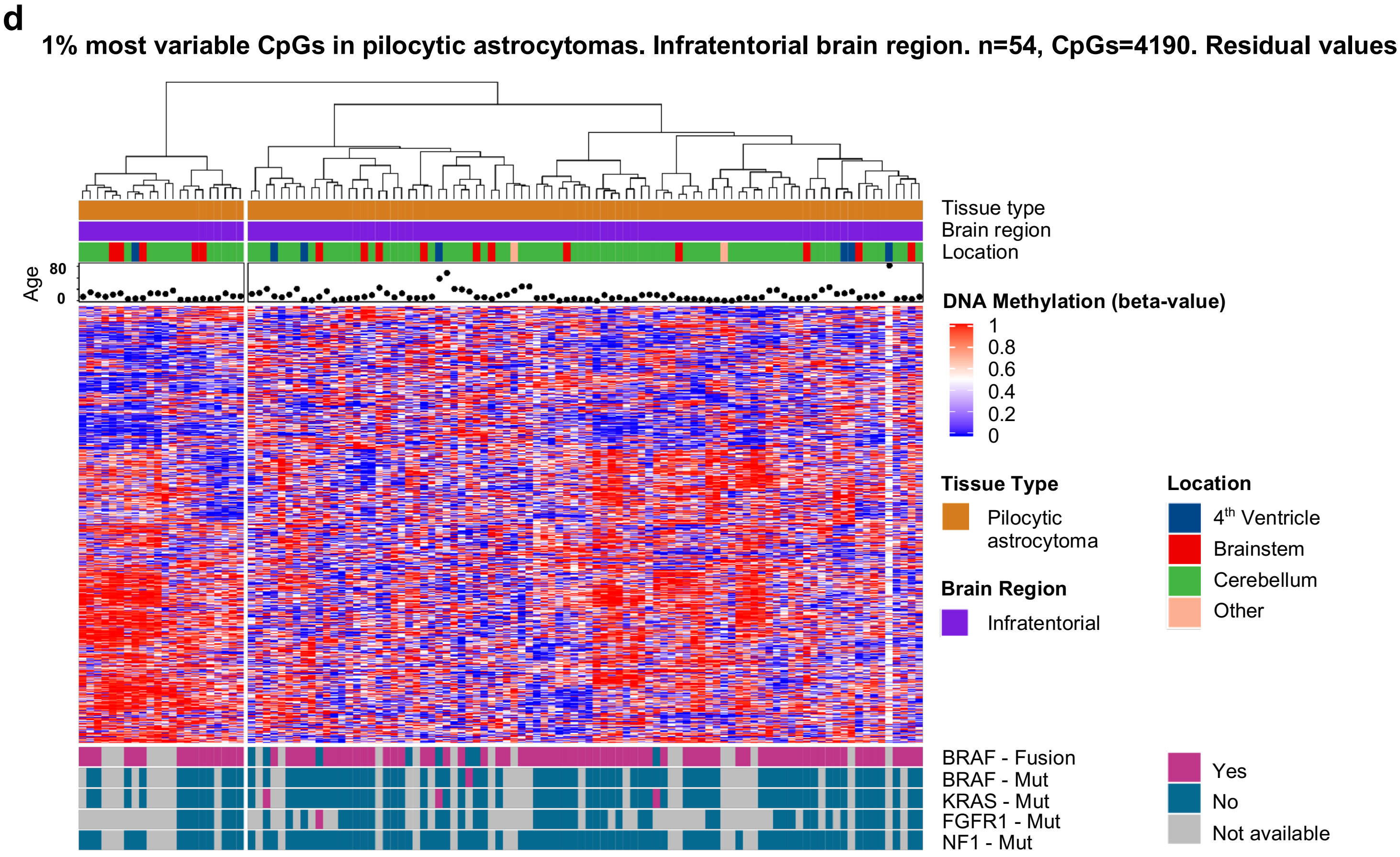

Supplementary Figure 8. Hierarchical clustering for supratentorial (a, b) and infratentorial (c, d) brain regions incorporating the tumor cohort based on the tumor most variable one percent of fitted CpGs or residual CpGs as indicated on the panels (variability measure: standard deviation, distance measure: pearson, agglomeration: ward.D).

Supplementary Figure 9

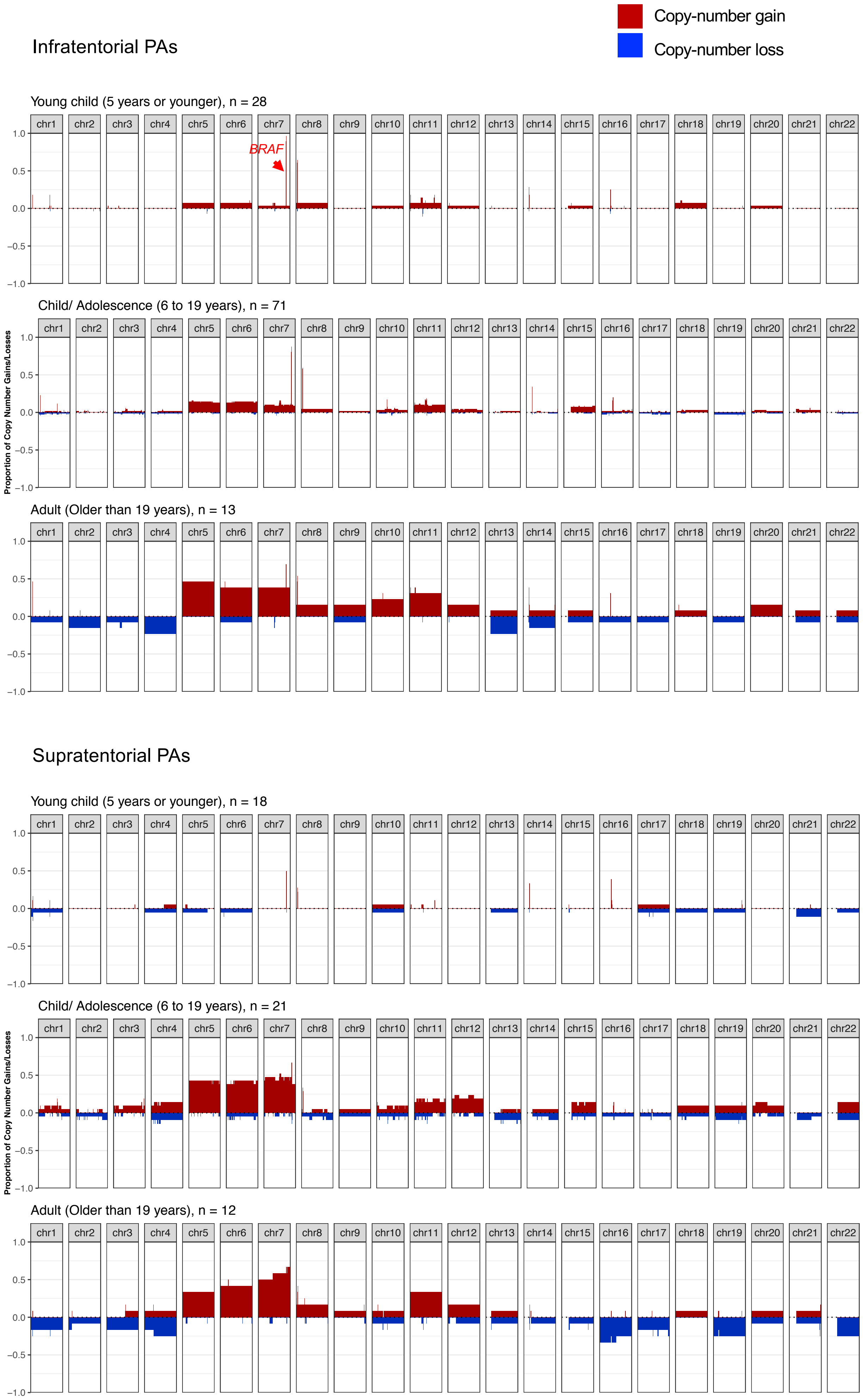

Frequency of DNA copy-number abberations based on methylation data for infratentorial and supratentorial pilocytic asrocytomas stratified according to age. Losses are shown in blue and gains in red.

Supplementary Figure 10

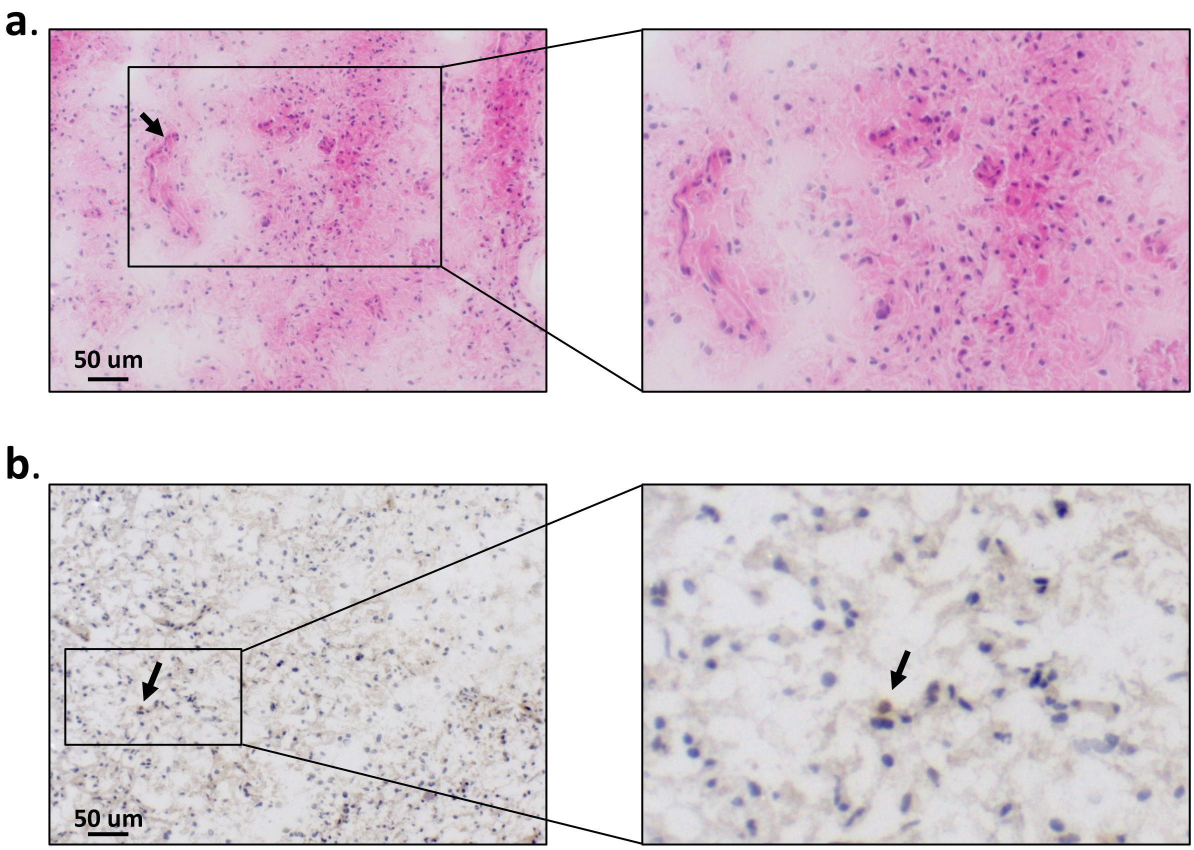

Supplementary Figure 10. Histopathological characterization of fresh frozen pilocytic astrocytoma samples used for the nuclei extractions and carbon dating. **a.** Representative hematoxylin and eosin stain showing one of the characteristic features of pilocytic astrocytomas, namely Rosenthal fibers (black arrow). **b.** Representative image illustrating low proliferative Ki67 index, typical for pilocytic astrocytomas. Ki67 positive cell (brown staining) is indicated by the black arrow. The blue staining shows the hematoxylin counterstain.

Supplementary Figure 11

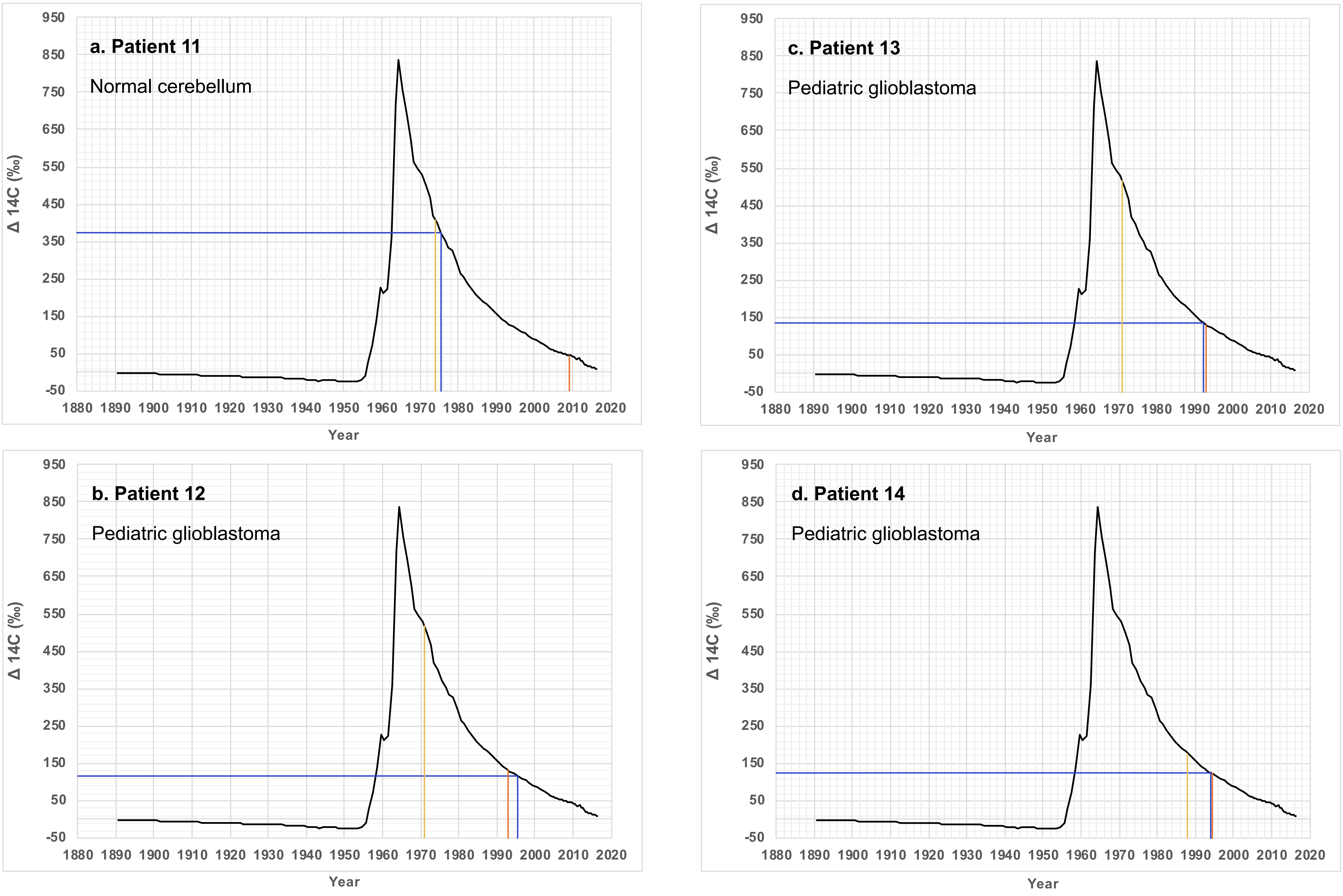

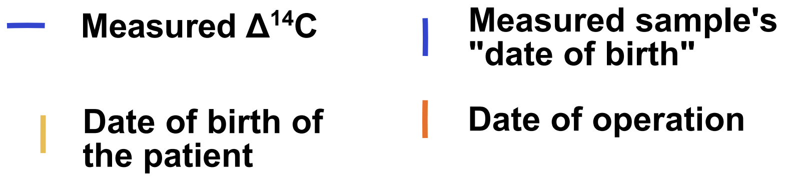

Supplementary Figure 11. The results of retrospective ^{14}C dating of pediatric glioblastoma samples. The black curve shows the values of the ^{14}C concentration in the atmosphere of the Northern hemisphere from 1890. Using this curve and the experimentally determined **Δ^{14}**C for the samples **a,** normal cerebellum, **b-d,** pediatric glioblastoma, the date when the cells from the respective tissue sample were generated can be calculated (dark blue lines). In addition, for each donor the date of birth of the individual (yellow) and the date of surgery (orange, identical to the date of sample collection) are indicated. Further details on these samples can be found in Table 1: *panels **a-d** correspond to sample IDs 11-14, respectively*. ^{14}C levels from modern samples are by convention given in relation to a universal standard and corrected for radioactive decay, giving the **Δ**^{14}C value.
