# Supplementary figures and images for: The age of adult pilocytic astrocytoma cells
Source: Oncogene. 2021 Mar 17;40(16):2830–41. doi: 10.1038/s41388-021-01738-0 (PMC8062266; doi:10.1038/s41388-021-01738-0)

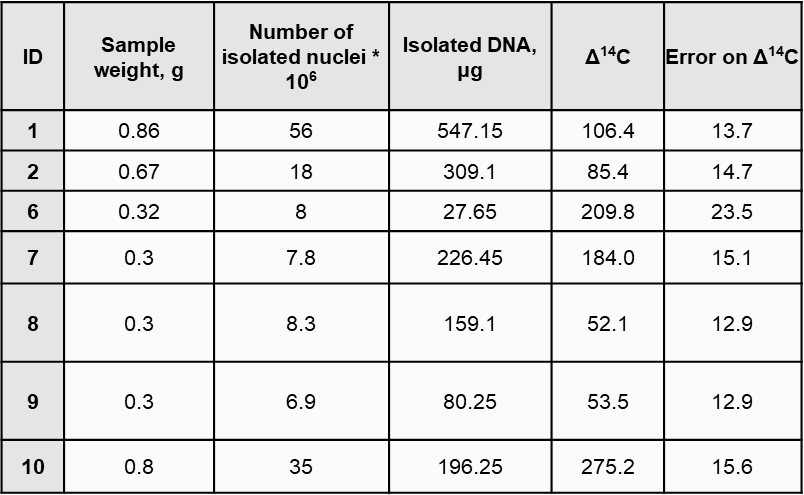


**Supplementary Table 4**. Technical details on samples used for the retrospective 14C dating (IDs 1, 2, 6-10).

Supplement: Supplementary file 5 — Supplementary Table 4 [file 41388_2021_1738_MOESM5_ESM.doc]
