# Supplementary material for: The age of adult pilocytic astrocytoma cells
Source: Oncogene. 2021 Mar 17;40(16):2830–41. doi: 10.1038/s41388-021-01738-0 (PMC8062266; doi:10.1038/s41388-021-01738-0)
Supplement: Supplementary file 6 — Supplementary Methods [file 41388_2021_1738_MOESM6_ESM.docx]

**Supplementary Methods**

**Analysis of incidence by age and primary site for PA**

The data used for the incidence analysis of pilocytic astrocytomas was provided by the Central Brain Tumor Registry of the United States (CBTRUS) 2012-2016. The CBTRUS dataset and standard analytical approached are described in detail in the CBTRUS Statistical Report: Primary Brain and Other Central Nervous System Tumors Diagnosed in the United States in 2012–2016.(Ostrom et al., 2019). The incidence rates of pilocytic astrocytoma were calculated per 100,000 by age and primary site. Age groups were selected according to the 2000 US Std Population standard (19 age groups - Census P25-1130). Primary site groupings were defined per the International Classification of Diseases Oncology version 3 (ICD-O 3) primary site coding as: infratentorial (Cerebellum C71.6, Brain Stem C71.7, Cranial nerves C72.4 acoustic nerve, Ventricle C71.5, Spinal structures C.72.0 – C72.1) and supratentorial (Cerebrum C71.0, Frontal lobe C71.1, Temporal lobe C71.2, Parietal lobe C71.3, Occipital lobe C71.4, Pineal gland C75.2, Cranial nerves (C72.2-C72.3): olfactory and optic, Pituitary gland and craniopharyngeal duct C75.1-C75.2, Meninges C70.9). The following primary sites were excluded from analysis: overlapping lesion of the brain C71.8, brain, NOS C71.9, cranial nerve, NOS C72.5. Statistics were not displayed due to fewer than 16 cases in some of the categories.

**Nuclei isolation for ^14^C content measurement**

Tissue samples for retrospective ^14^C dating were processed as described previously (Ernst et al., 2014). Shortly, specimens were thawed and homogenized on ice with a glass douncer after adding ice cold lysis buffer (0.32 M sucrose, 5 mM CaCl_2_, 3 mM magnesium acetate, 2.0 mM EDTA, 10 mM Tris-HCl [pH 8.0], 0.1% Triton X-100, 1 mM DTT). The volume of the buffer was calculated from the proportion: 10 ml per g of the tissue. The resulting homogenized tissue was passed through a 70 um cell strainer and further diluted by ice cold 1.8 M sucrose solution, 20 ml per each g of the original tissue. The obtained solution was mixed and equally layered to a cushion of 10 ml of 1.8 M sucrose solution in a microcentrifuge tube (pre-coated with BSA, 10g in 1l PBS). Using the sucrose density gradient of these layers, the nuclei were separated by centrifugation at 13000 rpm for 140 min at 4°C. The supernatant was carefully aspirated and tube sides cleaned with filter paper to remove myelin. The pellet of nuclei was then resuspended with 300 ul of cold nuclei suspension buffer (10 mM Tris [pH 7.2], 2 mM MgCl2, 70 mM KCl, and 15% sucrose), transferred to an appropriate vial and an aliquote was taken to determine the number of nuclei. Prepared nuclei were stored at -80°C.

**DNA extraction**

DNA extraction from the isolated nuclei for the following ^14^C content measurement was performed as previously described (Spalding et al., 2005). Briefly, nuclei were suspended in 1 ml of solution containing 1% of SDS, 5 mM EDTA-NA_2_, 10 mM Tris-HCl (pH8.0) and 6 μl protein K solution (20 mg/ml, Invitrogen). The sample solution was mixed by gently tapping the sample tube a few times and then incubated at 65°C overnight. RNase cocktail (3 μl, Ambion, #2286) was then added to each sample and incubated for 45 min at 65°C. NaCl (500 μl, 5M) was then added to the solution, vortexed for 15 s and centrifuged at 13 000 rpm for 3 min. The supernatant was carefully transferred to 15 ml glass vials. Three times the volume of ethanol (95%) was added to the vials, which were gently inverted a few times until a DNA precipitate was visible. The DNA pellet was washed three times in ethanol solution (70% ethanol, 0.5 M NaCl) for 15 min. Finally, the DNA pellet was transferred to a 2 ml glass vial and resuspended in 500 μl DNase/RNase free water (GIBCO/Invitrogen) and incubated overnight at 65°C. The DNA was quantified using a Nanodrop 2000 (ThermoScientific) and only samples within a defined range (260/280: 1.8-2.0; 260/230: 2.0-2.4) were included for ^14^C analysis.

**Measurement error and purity correction of ^14^C Values**

The AMS Δ^14^C measurement errors are reported in Supplementary Table 4. All ^14^C data are reported as decay-corrected Δ ^14^C or fraction modern (F^14^C).

Since the samples 1-7 (Table 1) corresponding to adult and pediatric pilocytic astrocytomas were not sorted, they contained 5-30 % of non-target cells which could be of later or earlier origin compared to the tumor cells based on their turnover rate. We accounted for this impurity considering that tumor cell content identification might have 10% points methodological error in addition. To do so, we assumed the earliest possible and latest possible age of the normal cells (birth, time of operation) which would represent the worst-case scenarios.

Δ ^14^C Tu = Δ ^14^C obs /TC - Δ ^14^C normal/TC + Δ ^14^C normal

Where

Δ ^14^C Tu = possible Δ ^14^C of the tumor in the hypothetical scenario that all non-tumor cells are either of the earliest possible or latest possible age

Δ ^14^C obs= observed measured Δ ^14^C of the sample

Δ ^14^C normal = Δ ^14^C of the normal cells present in the sample in the hypothetical scenario that all non-tumor cells are either of the earliest possible (sample collection date) or latest possible (donor’s date of birth) age

TC = tumor cell content

Errors were calculated using propagation of error and approximation to a first-order Taylor series expansion.

**Statistics**

Linear regression analysis was performed to assess the difference between age groups defined by hierarchical cluster analysis. Cluster groups as well as tissue type were dummy coded, with cluster 4 and control tissue as reference group. Tumor immune infiltration was examined in silico using the R-package EpiDISH using the CIBERSORT algorithm and the default immune reference set. The code was adjusted to return absolute values instead of fractions. To investigate the influence of immune infiltration and age on the methylation profile of PA, a gaussian mixture model, stratified by brain compartment, was estimated. Age as well as abundance of B-cells, monocytes, eosinophils, CD4+ T-cells and natural killer cells were used as independent variables. To project unit-interval bound beta methylation values to the real number space, m-value transformation was used. To allow for multi-modal distributions, mixture models with up to four combined distributions were fitted. The model was fitted for each CpG position independently and AIC filtering was used to assess the best performing mixture model. Tests were considered significant, if the test statistic was equal or smaller than 0.05.
